# A Draft of the Human Septin Interactome

**DOI:** 10.1371/journal.pone.0013799

**Published:** 2010-11-02

**Authors:** Marcel Nakahira, Joci Neuby Alves Macedo, Thiago Vargas Seraphim, Nayara Cavalcante, Tatiana A. C. B. Souza, Julio Cesar Pissuti Damalio, Luis Fernando Reyes, Eliana M. Assmann, Marcos R. Alborghetti, Richard C. Garratt, Ana Paula U. Araujo, Nilson I. T. Zanchin, João A. R. G. Barbosa, Jörg Kobarg

**Affiliations:** 1 Laboratório Nacional de Biociências, Centro Nacional de Pesquisa em Energia e Materiais, Campinas, Brasil; 2 Departamento de Bioquímica-Programa de Pós-graduação em Biologia Funcional e Molecular, Universidade Estadual de Campinas, Campinas, Brasil; 3 Centro de Biotecnologia Molecular Estrutural, Universidade de São Paulo, São Carlos, Brasil; 4 Centro de Biologia Molecular e Engenharia Genética e Faculdade de Ciências Aplicadas, Universidade Estadual de Campinas, Campinas, Brasil; 5 Pós-Graduação em Ciências Genômicas e Biotecnologia, Universidade Católica de Brasília, Brasília, Brasil; Universidade de São Paulo, Brazil

## Abstract

**Background:**

Septins belong to the GTPase superclass of proteins and have been functionally implicated in cytokinesis and the maintenance of cellular morphology. They are found in all eukaryotes, except in plants. In mammals, 14 septins have been described that can be divided into four groups. It has been shown that mammalian septins can engage in homo- and heterooligomeric assemblies, in the form of filaments, which have as a basic unit a hetero-trimeric core. In addition, it has been speculated that the septin filaments may serve as scaffolds for the recruitment of additional proteins.

**Methodology/Principal Findings:**

Here, we performed yeast two-hybrid screens with human septins 1–10, which include representatives of all four septin groups. Among the interactors detected, we found predominantly other septins, confirming the tendency of septins to engage in the formation of homo- and heteropolymeric filaments.

**Conclusions/Significance:**

If we take as reference the reported arrangement of the septins 2, 6 and 7 within the heterofilament, (7-6-2-2-6-7), we note that the majority of the observed interactions respect the “group rule”, i.e. members of the same group (e.g. 6, 8, 10 and 11) can replace each other in the specific position along the heterofilament. Septins of the SEPT6 group preferentially interacted with septins of the SEPT2 group (p<0.001), SEPT3 group (p<0.001) and SEPT7 group (p<0.001). SEPT2 type septins preferentially interacted with septins of the SEPT6 group (p<0.001) aside from being the only septin group which interacted with members of its own group. Finally, septins of the SEPT3 group interacted preferentially with septins of the SEPT7 group (p<0.001). Furthermore, we found non-septin interactors which can be functionally attributed to a variety of different cellular activities, including: ubiquitin/sumoylation cycles, microtubular transport and motor activities, cell division and the cell cycle, cell motility, protein phosphorylation/signaling, endocytosis, and apoptosis.

## Introduction

Septins belong to the GTPase superclass of P-loop NTPases. Specifically, they belong to the TRAFAC class which includes the Ras-like superfamily, Myosin-kinesin superfamily, and translation factor superfamily [1]. They are found in all eukaryotes, from yeast to mammals, except in higher plants [2] but the number of septin genes found in different species varies considerably. For example *Saccharomyces cerevisiae*, has seven septin genes (Cdc3, Cdc10. Cdc11. Cdc12, Shs1, Spr28, Spr3), whilst *Caenorhabditis elegans* has only two (Unc59 and Unc61), *Drosophila melanogaster* five (Pnut, Sep1, Sep2, Sep4. Sep5), and *Mus musculus* thirteen (Sept1-Sept9, Sept10a, Sept10b, Sept11, Sept12) [2]–[4]. In humans, so far 14 septin genes have been reported. However, many of them present several splice variants, which are designated by a nomenclature in which a variant of SEPT6, for example, would be indicated as SEPT6_v1. SEPT9 is particularly variable in this respect [5].

Mammalian septins have been classified into 4 different groups, based on their amino acid sequences. At present several different nomenclatures exist [2], [6]–[8]. The simplest of these makes use of a reference septin for each group. Thus, the SEPT2 group (also called group 2B [2] or group III [7]) contains SEPT1, SEPT4 and SEPT5 as well as SEPT2. Similarly, the SETP3 group (alternatively called group 1A or group I) consists of SEPT3, SEPT9 and SEPT12 and, the SEPT6 group (also called group 1B or group II) contains: SEPT6, SEPT8, SEPT10, SEPT11 and SEPT14. The SEPT7 group, which includes in addition to SEPT7 also SEPT13 (group IV), is normally considered to form the fourth independent group. However, the latter two septins have sometimes been considered to form a sub-group of the SEPT2 group (part of group 2B) with which they are closely related phylogenetically [2]–[4].

Septins have molecular masses that vary between 30 and 65 kDa and present a high sequence identity in their conserved central GTPase domains, featuring typical Ras like GTPase motifs and P-loop signatures found in most GTPases [2], [6], [9]. This central GTP-binding domain generally also contains a short polybasic region prior to the P-loop (or Walker A box). This has been shown to bind to phospholipids and may be responsible for mediating interactions with membranes [9]. The central domain is flanked by an amino-terminal domain which is variable in both sequence length and identity and a carboxy-terminal domain which also varies considerably from one septin to another, but frequently contains a coiled-coil region, possibly involved in mediating protein-protein interactions. In the case of mammalian septins, only members of the SEPT3 group lack the coiled-coil signature.

One of the most remarkable features of septins is their ability to engage in the formation of homo- and hetero-meric filaments, which seem to be important for mediating their cellular functions. Aside the expected and demonstrated roles of these filaments in intracellular transport processes and cellular movements, especially in the context of cell division, the septin filaments have been predicted to serve also as scaffolds for the docking of other regulatory or signaling proteins [10].

Several studies have now shown that the typical filamentous form of mammalian septins appears to systematically involve a heterotrimer as its core module [10]–[12]. The complexes described so far vary, however, in size and composition. Biochemical studies reported to date suggest the existence of several different trimeric complexes including SEPT4-SEPT5-SEPT8 [7], SEPT7-SEPT11-SEPT9b [13], SEPT5-SEPT7-SEPT11 [14] and SEPT3-SEPT5-SEPT7 [15], [16]. The most well studied of all is that of human septins 2, 6 and 7 [17] which remains the only septin complex to have been solved crystallographically to date. Its structure, at 4.0 Å was reported together with that of a fragment of human SEPT2 which lacks 46 residues of the predicted coiled-coil region at the C-terminus [18]. These structures showed that the GTPase domain is responsible for polymerization and septin-septin interactions at the so called G and NC interfaces result in the assembly of linear non-polar polymers. More recently, the crystal structure of the GTP bound form of SEPT2 was reported at 2.9 Å resolution, revealing that GTP binding induces a conformational change in the switch regions directly affecting the G interface and indirectly, the NC interface [19]. Moreover, GTP binding/hydrolysis and the nature of the bound nucleotide influence the stability of the interface in the heterooligomeric and polymeric state, as well as filament assembly and disassembly.

Septins have been shown to be functionally involved in a diversity of processes ranging from cytokinesis [12], [20], cell membrane dynamics [21], [22], signal transduction cascades, cellular signaling events [6], [23], cell cycle control and others [1], [24]. Moreover, septins have been shown to regulate bacteria-host interactions [25] and to be important determinants for yeast virulence [26]–[28]. Finally, septin dysfunction has been associated with several human pathologies, including cancer [29], Parkinson's disease [30], Alzheimer's disease [31] and hereditary neuralgic amyotrophy [32].

Despite all the documented progress, the exact molecular mechanisms of the septins, and their cellular and physiological functions are still poorly understood. As a first step in approaching molecular and cellular functions a comprehensive description of the interacting protein partners for the septins would be very valuable. This is especially important if we consider the possible role of the different septin filaments as docking or scaffolding platforms to mediate additional new functions. Although some isolated attempts to identify septin interactors using the yeast two-hybrid system have been made to date for the individual septins 5, 8, 9 and 14, so far no large scale analysis has been attempted [7], [25], [33]–[35].

Therefore we set out to perform yeast-two hybrid screens of the human septins 1–10, representing all 4 septin groups. In summary, we found that all septins, except SEPT10, interacted predominantly with other septins, principally those from other groups. The only exception came from the members of the SEPT2 group, which also interacted with partners from the same group. Most, although not all, of the results were confirmative in a reciprocal sense. For example, SEPT3 when used as a bait molecule identified SEPT6 as a partner and when SEPT6 was used as the bait, SEPT3 was identified. Most interestingly, the results on the whole seemed to conform to the proposed trimeric arrangement in a group format. This means that if we organize the two trimers in the following arrangement: Group 3/7-6-2 - 2-6-3/7, we can assign the great majority of the individually found interactions and all pair wise group interactions in this model are covered by statistical data which demonstrate that the experimental observed distribution of the fished septin clones is not random. If this interpretation of the trimer arrangement and the “group rule” holds true through additional biochemical experiments we can propose a range of new possible septin trimers, whose existence in cells should be tested.

All septins also interacted with other non-septin proteins. Those common to several septins or septin groups can be largely attributed to the ubiquitin and sumoylation cycles, transport and motor activity, cell division/cell cycle, and protein phosphorylation. Novel individual septin or septin family specific functions include: apoptosis (SEPT3 and SEPT6), transcription (SEPT8 and SEPT7) and DNA repair and splicing (SEPT3), among others. Our data shed new light on septin function and provide a wealth of new information to form new hypotheses, especially with respect to septin trimer and filament formation and the association of septins within new cellular contexts. Future biochemical, structural and cellular functional studies are required to test the newly proposed hypotheses.

## Results and Discussion

### The interactions of septins with other septins

**Figure 1:**
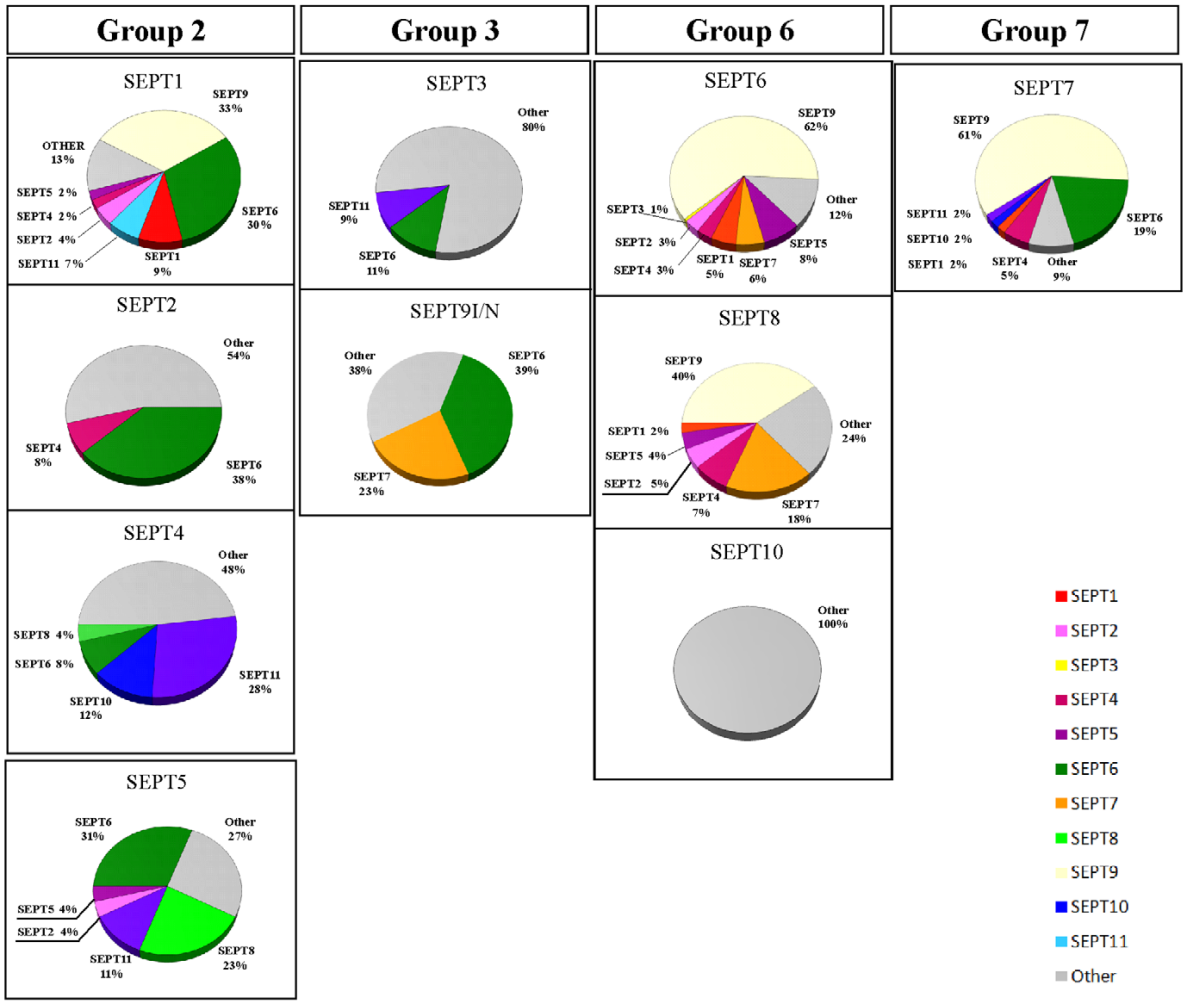
Summary of protein-protein interactions found for human septins 1-10. The values are given as a percentage of clones from the total number of confirmed interacting clones sequenced and identified. See color code for septin specification. Septins are grouped according to the four groups SEPT2, SEPT3, SEPT6 and SEPT7 from left to right.

Analysing the overall result of our initiative to characterize septin interacting proteins on a broad scale, one first is impressed by the large number of septin prey proteins that were discovered for all septin baits, with the sole exception of SEPT10. Although the latter did not interact with any other septin when used as bait, it was however selected in reasonable quantity by both SEPT4 (23% of the interacting septins) and to a lesser extent by SEPT7 (2% of the interacting septins). The complete data is given in resumed form in Table 1, Figure 1 and Figure S1. Please refer to Table S1 for complete primary data.

**Table 1:** Correlation of septin prey clones (columns) fished by a given bait septin (lines).

Prey: Bait:	1	2	4	5	6	8	10	11	3	9	7
1	3	2	1	1	**14**			3		15	
2			1		**5**						
4					**2**	**1**	3	7			
5		1		1	**8**	**6**		3			
6	**8**	**5**	**5**	**12**					**1**	**96**	**9**
8	**3**	**6**	**9**	**5**						49	22
10											
11											
3					**6**			5			
9					**5**						**3**
7	1		3		**11**		1	1		**34**	

The numbers are the number of identified clones. Bold and underlined numbers emphasizes those preys that also fished the corresponding prey when they were used as bait (reciprocal fishing). The baits and prey are listed regarding to membership in the four septin groups: 1,2,4,5 (group 2); 6,8,10,11 (group 6), 3,9 (group 3) and 7 (“group 7”).

Members of the SEPT6 group, with the exception of SEPT10, had a clear tendency to interact with other septins instead of with other non-septin proteins (Fig. 1). For example, SEPT6 had only 12% non-septin interactors and SEPT8 only 24%. A similar result is observed for SEPT7, for which only 9% of the identified interactors were non-septins. Several other septins, including SEPT2, SEPT4 and SEPT3, showed the opposite tendency and tended to interact with a relatively large number of non-septin partners. SEPT2 interacted with 54%, SEPT4 with 48% and SEPT3 with an impressive 80% of non-septin preys.

**Figure 2:**
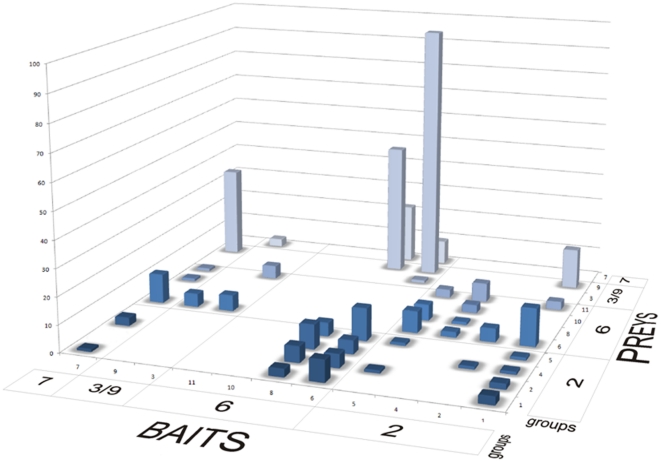
Three dimensional column diagram of the number of clones of septin prey proteins fished by septin bait proteins in group wise organization. Number of fished clones for each prey septin (Y-axis), was plotted against bait (X-axis) and prey septins (Z-axis), where the septins have been ordered in a group wise fashion (See Table S1 for raw data of clone numbers). The whole distribution is significantly (p = 0.0004998) different from a random distribution, as can also be verified visually, since the clone numbers group into “islands” in between various blank areas. When we compared groups in a one-to-one and reciprocal fashion we obtained significant p-values (p<0.001), suggesting a non-random distribution, for the following pairs of groups: group 2 vs. group 6, group 6 vs. group 3/9, group 6 vs. group 7, and group 3/9 vs. group 7. Furthermore, we can still analyze the occurrence of interactions among septins of the same group. The result in this case is obvious: members of groups 6, 3/9 and 7 never interacted with themselves or with other members of the same group. The only exception is group 2, which members tended to interact with other group 2 members. See also Fig. 3.

Interestingly, several septins showed a marked preference for a particular septin as partner from among those identified. SEPT2 predominantely interacted with SEPT6 (38%), SEPT9/SEPT9(1-269) mostly with SEPT6 (39%) and SEPT6, SEPT8 and SEPT7 all interacted predominantly with SEPT9 (62%, 40% and 61%, respectively). For the whole table of groups of baits vs. groups of prey septins we obtained with Fischeŕs exact test a p value of 0.0005, diagnosed as significant (Table S1). In other words, the data are far from being randomly distributed. This can also be seen directly from Fig. 2, where islands and clusters can be easily observed in between many blank regions.

Although there was not always a strict reciprocity in the sense that each bait which picked up a given septin prey was also found as a prey when the interacting septin was used as bait, several interesting tendencies can nevertheless, be detected. In Table 1 the number of fished septin clones is listed in accordance with their group membership which aids in emphasizing a certain clustering of blocks of interactions which can be readily observed (clone numbers in bold and underlined). Block 1 corresponds to the use of SEPT6 and SEPT8 as bait. Both these septins picked up predominantly septins of the SEPT2 group (Block1a) and members of group 3/9 and 7 (Block1b), but never septins of the same group (SEPT6). Block 2 refers to the SEPT2 group when used as bait, which preferentially picked up septins of the SEPT6 group (Block2a). Most interestingly, for both of these blocks there is also a considerable degree of reciprocity (bold underlined numbers in Table 1), which lends an additional degree of confidence to the results. For example bait SEPT6 fishes prey SEPT3/9/7 and in reverse bait septin 3/9/7 fished prey SEPT 6. A third block also corresponds to the use of the SEPT2 group septins as bait and which had a weak but consistent tendency to pick up preys of the same group: SEPT1 interacted with SEPT1/2/4/5; SEPT2 with SEPT4, and SEPT5 with SEPT2/5. The latter is the only example of self interactions among members of the same group, detected during this study. These intra-group interactions observed for the SEPT2 members are in stark contrast to what is observed for the SEPT6 group members and are probably pertinent to filament formation as described below.

### Comparison of the septin-septin interactions within the format of the septin trimer

**Figure 3:**
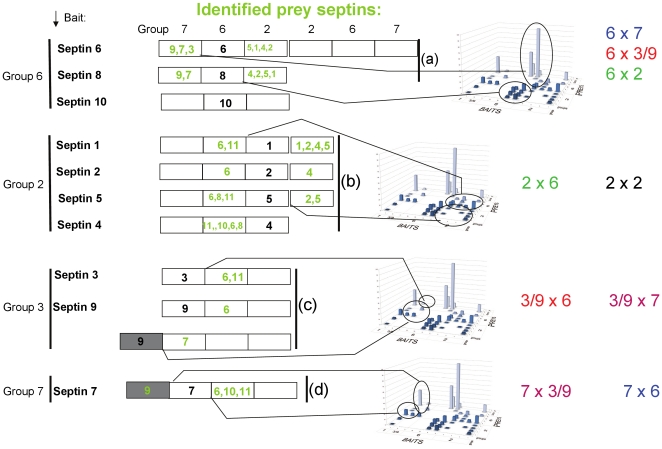
Representation of the two-hybrid septin-septin results in the light of the format of the trimer/hexamer SEPT7/SEPT6/SEPT2 – SEPT2/SEPT6/SEPT7 [18]. Assuming that members of the same group may serve as substitutes [6] and taking into account the structural arrangement found for the crystal of the trimer/hexamer SEPT 7/6/2, interesting observations can be made. The bait septins employed in the two-hybrid system are given on the left. In green in the schematic figure the preferentially found prey septins are indicated and assigned to likely positions in the hexamer scheme. The three dimensional column diagrams on the right refer to Fig. 2. The data that differ significantly from a random distribution have been circled to indicate the experimental basis on which each structural arrangement (monomer interfaces) is based. From top to bottom: (a) is based on the following statistical comparison: group 6 vs. group 2 (no random distribution: p< 0.001), group 6 vs. group 3/9 (p<0.001), group 6 vs. 7 (p<0.001). (b): group 2 vs. group 6 (p<0.001), (c): group 3/9 vs. group 6 (p<0.001), group 7 vs. group 9/3 (p<0.001), (d): group 7 vs. group 6 (p<0.001), group 7 vs. group 9/3 (p<0.001). None of the septins fished members of its own family, except group two members (b). The group pairings with statistically significant clone distributions were indicated at the right side of the figure (e.g. 6×7, 3/9×6 etc.). By comparison of the letters color codes it can be seen that all of these group pairs were reciprocal. For example bait septin 6 group fished group 2 septins (a) and vice versa (b) (p <0.001). As initially proposed by Kinoshita there may be substitutions among different members of the same septin group. The sequence of listed septins from left to right reflects a descending order of frequency of clones with septins that were found to interact (e.g. for SEPT6 bait: 9,3,7 and 5,1,4,2).

The interaction results described in the previous paragraph gain an interesting new perspective when analyzed in the light of the structural data obtained for the septin trimer 7-6-2 [18] and further taking into consideration Kinoshitás prediction [6] that in the trimer format septins from within a given group may substitutes for one another (Figs. 2 and 3).

Analysis of the results for the SEPT6 group members as baits, given the known arrangement of the trimer, reveals interesting new insights. SEPT6 occupies the central position in the trimer structure and Kinoshita's prediction suggests that other members of the group, namely SEPT8, SEPT10, SEPT11 and SEPT14 should be competent substitutes at this position of the filament. Hence, the SEPT6 group members could be predicted to be sandwiched between SEPT2 and SEPT7 group members. Based on this assumption it would be expected to find them largely interacting with septins from the latter two groups but never with members of their own. The number of clones corresponding to an interaction between SEPT6 group members and SEPT2 group members is statistically significant (p<0.001) as is that between SEPT6 and SEPT3 group members and between SEPT6 and SEPT7 group members. [Fig. 3, (a)] (Please refer to Materials and Methods section for details of the statistical analysis).

The only exception is SEPT10, which seems to behave differently altogether, since, when used as bait, we did not identify any septin partners at all. However, consistent with the above scenario, SEPT10 was identified as a binding partner of both SEPT4 (a SEPT2 group member) and SEPT7 in the reverse experiments. In summary these findings are entirely in accordance with what would be expected from the trimer model and allow us to propose new possible trimer configurations, which can be summarized as 7-8-1/2/4/5 or 7-6-1/4/5. Although we are unaware of any direct experimental evidence reporting these particular trimeric arrangements it is worth mentioning that a complex of SEPT5, SEPT7 and SEPT11 (a very close relative of SEPT6) has been reported recently [14]. The physiological relevance of such potential complexes clearly depends on other factors such as the tissue specific expression of different septins and their splice variants [36].

Notably, of all the 10 septins analyzed, SEPT6 followed by SEPT8 showed both the largest number of interacting clones as well as the greatest number of different identified candidate interactors. Since the majority of clones found to interact represented other septins (88% and 76% for SEPT 6 and 8, respectively), this may reflect the fact that the members of this group always occupy the central position of the filament's trimeric unit. In co-purification studies we were able to demonstrate that GST-SEPT6 co-purified with septins 1, 2, 3, 5, 7 and 9, when both are expressed together in *E. coli*, thereby confirming the majority of the interactions given in Table 1 (Nakahira et al., unpublished observations). It has been recently shown that septins of this group (SEPT 6, 8 and 11) show a lower rate of GTP hydrolysis when compared to SEPT2 (Souza et al., unpublished observation). Most interestingly, septins of this group have the key Ser residue in the G1 motif substituted by a Thr residue, possibly indicating why the septins of this group have a less efficient rate of hydrolysis. This speculation has some support in the finding that in the crystal structure of the 7-6-2 hetero-trimer GDP is observed bound to SEPT2 and SEPT7 but GTP to SEPT6 [18].

A very interesting result was obtained when the SEPT2 group members were used as bait [Fig. 2, Fig. 3(b)]. In this case the majority of preys belong to the SEPT6 group (46, 38, 52 and 65% respectively, for baits SEPT1, SEPT2, SEPT4 and SEPT5). This is consistent with what is expected from the canonical 7-6-2 trimer, where SEPT2 makes direct contact with SEPT6. Again the preference of SEPT2 group members for partners from SEPT6 group is statistically significant (p<0.001, Fig. 3b).

**Figure 4:**
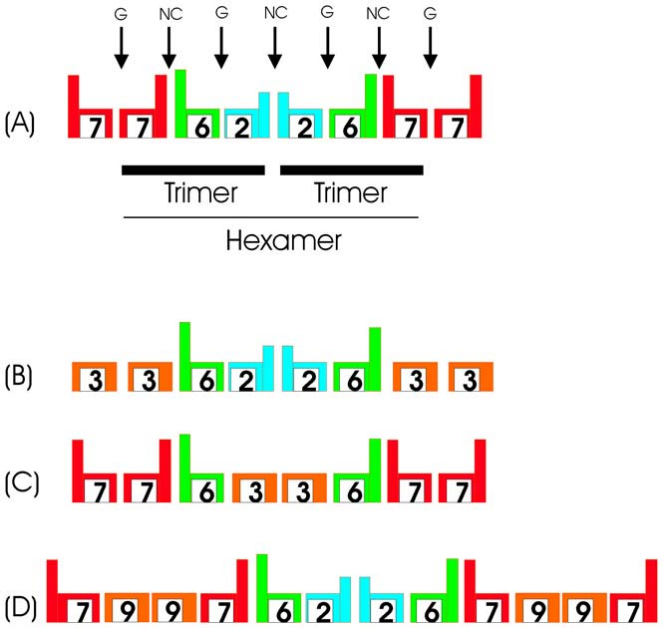
Schematic representation of possible septin-septin interactions within putative filaments, based on a combination of our yeast two hybrid assay's data and previously published data. (A) the canonical or standard filament taken from the crystal structure of the 7-6-2 complex [18] showing the trimeric and hexameric cores and the NC and G interface. The 7-6 dimer is believed to be stabilized by a long coiled coil at an NC interface, whilst the 2-2 dimer is similarly stabilized by a short coiled coil. (B) possible arrangement for a filament composed of members from the SEPT3, SEPT6 and SEPT2 groups. SEPT3 group contains both septin 3 and 9. This arrangement would leave the SEPT6 coiled coil unpaired and “free” for interaction with other coiled-coil containing proteins (see text for details). (C) possible arrangement for a filament composed of members from the SEPT3, SEPT6 and SEPT7 groups. (D) possible position for SEPT9 compatible with the observed yeast two-hybrid data and data from the literature [38] (see also Fig. 3c,d).

A small but consistent fraction of the preys belonged to the SEPT2 group were found as partners of septins of the same group (8%, for each of SEPT1, SEPT2 and SEPT5, when used as bait). This makes SEPT2 group members an exception as the only septins which interacted with members of their own group and may reflect the fact that the first stage of polymerization appears to be the formation of hexamers (or dimers of trimers) in which one copy of SEPT2 makes contact with a second via what has been called an NC interface [18] (Fig. 4A). Furthermore, SEPT2 when expressed and purified as a dimer, although curiously this appears to use the G interface rather than NC [19]. Together, this further suggests that SEPT1, 4, and 5 can substitute SEPT2 in the trimer format and reinforces the proposed 7-6-1/4/5 combinations described above. It is interesting to note that none of the SEPT2 group septins picked up SEPT7, as would be also expected, given that there is no direct contact made between them in the trimer. However, during the reverse experiment SEPT7 identified a small number (7%) of SEPT2 group clones. These may correspond to real hits corresponding to hetero-polymers of different arrangement or may be artifacts. It is known for example that SEPT2 alone is able to form continuous polymers similar to those seen in the 7-6-2 complex, in which it makes use of both G and NC interfaces. The G interface may be promiscuous in that it is not observed in the hetero-polymer and similar promiscuity may be observed with other septins leading to possible artifacts. What is noteworthy, is the fact that the vast majority of observed interactions are consistent with the 7-6-2 filament and with Kinoshita's conjecture [6].

Besides identifying the expected binding partners predicted from the trimeric arrangement, SEPT6 and SEPT8 also interacted with SEPT3 group members, notably SEPT9 (Fig. 3a, p<0.001). Furthermore, in the reverse sense, when SEPT3 and SEPT9 were used as bait, they preferentially fished out preys belonging to the SEPT6 group (39% in the case of SEPT9 for example, Fig. 3c, p<0.001), consistent with previous studies [13], [37]. Since the SEPT3 group is not present in the canonical 7-6-2 trimer, this raises the intriguing question of how they may participate in filament formation. Since SEPT6 occupies the central position, one possibility is that the SEPT3 group members may replace either SEPT2 or SEPT7. If they were to occupy the position normally occupied by SEPT7, this would lead to 3-6-2 and 9-6-2 as possible new trimeric arrangements (Fig. 3c, Fig. 4B). However, this would be incompatible with the coiled coil C-termini which are expected to project laterally, at a 90 degree angle, from the filament (Fig. 4A,B), since it would leave the SEPT6 coiled coil unpaired. On the other hand the coiled-coil pairing may have only a stabilizing function, since we know that the filament formation is primarily based on GTPase domain interactions [18], [19]. An alternative would be for the SEPT3 group to substitute SEPT2 at the center of hexameric unit (Fig. 4C). This would lead to combinations of the type 7-6-3 or 7-6-9. Included in the latter group is the combination 7-11-9b which has been experimentally reported [13].

Although we did not observe homotypical interactions of the type: septins 7×7, 3×3 or 9×9 in our yeast two hybrid screens the above mentioned models (Fig. 4) synthesize our data with those already described in the literature (e.g. 7×7, 9×9). An intriguing possibility is that we never observed these homotypical septin interactions in our yeast two hybrid assay, because the latter is limited to one-to-one interactions among individual septins. It may be possible that 7×7 and 9×9 predominantly interact in the context of trimer-trimer or hexamer-hexamer interactions at the final stage of filament formation. This may be due to conformational changes which could occur at the septins 3, 7 and 9 when the hexamer units are formed. Septin 7 in a hexamer context would therefore gain then the capacity to interact with itself. Either way, these potential new filaments need to be verified *in vivo* and evaluated by further biochemical studies *in vitro*.

SEPT9 and SEPT7 identified one another as mutual partners at very high percentages. 23% of the prey clones identified by SEPT9 corresponded to SEPT7, whilst in the reverse direction the rate was even higher (61% of septin clones, Fig. 1). Again this distribution is significantly different from a random distribution (Fig. 3c,d), and the preferential interaction was reciprocal: septin bait 7 fished septin 9 (p<0.001) and septin bait 9 fished septin 7 prey (p<0.001). These results strongly suggest a physiological significance and possibly imply filament assembly, which is different from the canonical 7-6-2 arrangement. Surka and coworkers [38] described the immunoprecipitation of a complex containing septins 2, 6, 7 and 9 from HeLa cells, therefore suggesting that septin complexes may involve more than three components. This was confirmed more recently by Mostowy and coworkers [25] in which SEPT11 was replaced by SEPT6. A possible arrangement of SEPT7 and 9 in the filament is given in Figure 4D, where our own data are combined with data from the literature.

The SEPT3 members tested, especially SEPT3 itself, showed the lowest number of interactions with other septins but a relatively large number of interactions with other proteins (80%, Fig. 1). SEPT3 is highly expressed in neurons [8], which may imply a specialized function in these cells that may not be limited to septin filament formation, although it has been found in complex with SEPT6 members [13], [37]. SEPT9, on the other hand, is expressed ubiquitously, but occurs in many different variants in different tissues [8], [36]. Full length SEPT9 interacted only with SEPT6 and SEPT7, but its N-terminal region used alone as bait, only picked up non-septin proteins as interaction partners (see discussion below). Since SEPT9 lacks the C-terminal coiled coil domain, its interaction with other septins is likely to occur via the GTPase domain.

Besides the foregoing discussion SEPT7 also detected some clones of SEPT4 and SEPT1 (together 7%). These are not anticipated by the canonical 7-6-2 filament and may suggest a tendency to participate in other trimeric or even dimeric or tetrameric assemblies upon multimerization or filament formation. SEPT7 is expressed in most tissues and is therefore expected to be more involved in basic processes such as cell division. It belongs to the group of septins with the fewest members (SEPT7 and 13, only) and is present in almost all of the heterofilaments described to date. It may therefore turn out to be a fundamental element for filament formation.

SEPT11 (which is very similar to SEPT6), although not used as a bait by us here, was found as a prey in significant proportions when SEPT1, 4, 5 and 3 were used as bait and in relatively smaller proportion in the case of SEPT5 (Fig. 1). These interactions are consistent with the canonical filament where SEPT11 would occupy the central position of the trimeric unit. Curiously, neither septins 12, 13 nor 14 were ever picked up by any of our bait septins. This may either suggest that they are poorly expressed in the libraries tested or that these septins, like SEPT10 have no strong tendency to interact with other septins.

### Common non septin-interactors

**Figure 5:**
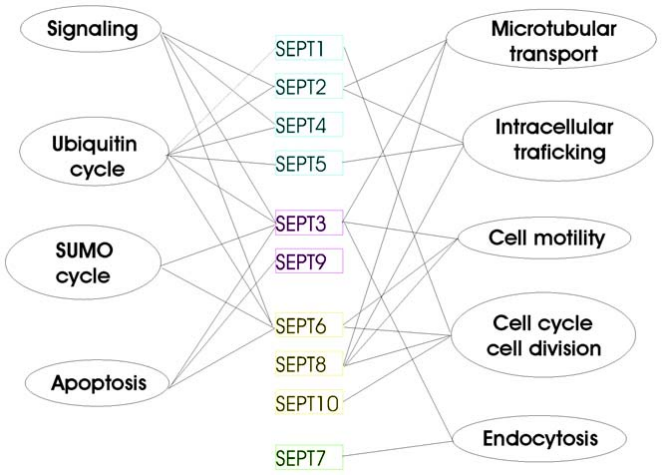
Non-septin interactors of the septins 1-10 grouped by functional categories. The group wise clustering of functional contexts is evident. The most predominant functions are emphasized only in order not to pollute the figure with excessive information. Septins are shown clustered into the four groups: SEPT2, SEPT3, SEPT6 and SEPT7 from top to bottom in different colors.

Aside the many septins found to interact with most of our baits, we observed varying quantities of non-septin proteins as interaction partners in all cases. These belonged to both structural and functional classes of proteins (Table 2, Figure S1). Most interestingly, some of them were repeatedly found with several different septins (e.g. UBE2I, SUMO or PIAS). In fact the latter proteins, functionally associated with the ubiquitin and sumo-cycles were found as interactors for all septins except SEPT7, SEPT9 and SEPT10. This indicated that at all members of the SEPT2 group and at least some members of the SEPT3 and SEPT6 groups have a propensity to interact with proteins from the sumo- and ubiquitin-cycles and seems to suggest possible protein degradation pathways relevant for human septins (Fig. 5). Furthermore, these findings show that the sumoylation process may be relevant for the regulation of septin functions, since SEPT3, SEPT6 and SEPT8 all picked up SUMO1 and/or the Sumo ligase PIAS3. Sumoylation of proteins has been shown to regulate the assembly and disassembly of protein complexes, their localization, stability and various other functions [39]–[41]. Previous data from the literature have demonstrated that yeast septins interact with SUMO and it has been suggested that sumoylation may be key for assembly of human septin filaments [42]. In yeast the septin ring formation depends critically on septin sumoylation [42]–[44].

**Table 2:** Summary table of all septin interacting proteins identified and their functional assignments.

Septin bait	Group	Septin preys (In order of frequency)	Predominant septin prey group	Non-septin preys in order of frequency (shared preys)	Functions of non-septins	Overlapping functions					
**1**	**2**	**Septin 9,6,11,1,2,4,5**	**Group 6**	**UBE2I, CEP110, SKA1**	Protein degradation, cell division					C	U
**2**	**2**	**Septin 6, 4**	**Group 6**	ANKZF1, DCTN2, CCDC45, PC-S/K-I, MAP3K12, ribosomal S6 Kinase like, **UBE2I**	Transport, Motor activity, microtubule-based process, kinase	T	M	P		C	U
**4**	**2**	**Septin 11, 10, 6, 8**	**Group6**	**UBE2I**, VEGFR-1, CASC3	Phosphorylation/Tyrosine kinase receptor, Protein degradation			P		C	U
**5**	**2**	**Septin 6, 8, 11, 5, 2**	**Group6**	**UBE2I**, SNX6	Protein degradation, Intracellular Trafficking	T				C	U
**9 (9N)**	**3 (3/9)**	**Septin 6, 7**	**Group 6**	FLNA, SH3KBP1	Cytoskeletal organization, apoptosis	T				C	
**3**	**3 (3/9)**	**Septin 6, 11**	**Group 6**	**UBE2I**, **CASP8AP2**, **PIAS3**, TDG, ABCB10, ACTB, EIF 4A, EXOSC9, GABA-RAPL2, HNRNPH3, MYO1B, PLK2, PRDX2, RPL14, RPS24, STMN2, **SUMO1**, TMEM93, IFT27, TDG	Protein degradation, Cell motility, Apoptosis, DNA repair, SUMO ligase, Neuron differentiation, Splicing process, Phosphorylation, Translation, Signal transduction, kinase, Endocytosis	T	M	P	G	C	U
**6**	**6**	**Septin 9, 5, 7, 1, 2, 4, 3**	**Group 3**	**UBE2I, SUMO1,** **CASP8AP2**, **PIAS3**, TOPORS, ACTR2, HIPK3, MDH1	Apoptosis, Cell cycle, SUMO cycle activity, Protein modification process, Ubiquitin cycle, Cell motility, Cell division, Protein degradation, kinase			P		C	U
**8**	**6**	**Septin 9, 7, 4, 2, 5, 1**	**Group 3**	C1QBP, CERCAM, CENP-F, SH2B3, UFD1L, CAPRIN1, ERP29, FAM89B, HDAC11, KIF14, LCP1, LMNB1, PBXIP1, **PIAS3**, SMARCC2, **ZNF451**	Immune response, Cell division, Cell adhesion, cell motility, SUMO ligase activity, Cell cycle, intracellular protein transport, Transcription, microtubule motor activity, Cell differenciation, ubiquitin cycle	T	M			C	U
**10**	**6**	**–**		PLZF-ZBTB16	cell cycle, transcription					C	
**7**	**7**	**Septin 9, 6, 4, 1, 10, 11**	**Group 3**	RALBP1, ANKRD12, **ZNF451**	Endocytosis, Transcriptional				G	C	

Overlapping functions: T =  Transport, endocytosis and cytoskeleton, M =  Motoractivity, P =  Phosphorylation, G =  GAP Ras, C =  Cell division, Cell cycle, U =  Ubiquitin / Sumo cycles (Pias, Ube2I).

Further functional contexts which are common to more than one septin or even to more than one septin group include: (a) Microtubular transport/intracellular trafficking/endocytosis (septins 2, 3, 5, 7 and 8); (b) Cell motility (septins 3, 6 and 8); (c) cell division/cell cycle (septins 1, 6, 8 and 10); (d) apoptosis (septins 3, 9 and 6) and (e) regulation through kinases/phosphorylation (signaling) (septins 2, 3, 4 and 6) (Table 2, Figure 5).

### Selected non-septin interactors

Some of the septins used as baits interacted with non-septin proteins or members of the same family of proteins that have been previously reported to interact with another septin. For example, the protein CENP-F was identified as an interactor of SEPT8 (Table 2). Interestingly, SEPT7 has been previously found to interact with CENP-E [45]. In that case it was reported that interference with SEPT7 iRNA prevents the correct localization of CENP-E to the kinetochore and abnormal chromosomal segregation [45]. Based on these and other experiments it was established that SEPT7 and CENP-E form an interacting pair in cells and that this interaction is not only important for the septin filament assembly but also for the correct formation of the kinetochore complex and is therefore essential for the mitotic spindle checkpoint [12], [45]–[48]. We may speculate that another such pair of interactors may be the proteins SEPT8 and CENP-F, whose interaction we describe here for the first time. This may suggest that SEPT8 in addition to other previously reported septins (SEPT2, SEPT7), is important for chromosome segregation and mitotic progression. Since this particular combination of septins (SEPT 7-8-2, Fig. 4) is expected to form viable filaments (see above discussion), this may imply that complex formation is relevant for fulfilling this physiological role.

Another individual interaction found, which merits discussion is that of SEPT5 and SNX6, since this may involve a coiled-coil interaction between the C-terminal domain of SEPT5 and one or both of the coiled-coil regions present in the C-terminal region of SNX6 [49], [50]. Coiled-coil domains (CC) are predicted to occur in up to 10% of eukaryotic proteins and are related to a wide array of different functions but serve predominantly to promote protein-protein interaction [51], [52]. SNX6 contains a Phox (PX) domain involved in phosphoinositol binding and members of the SNX family of proteins, like septins, are involved in intracellular trafficking. SNX members have been found in oligomeric complexes with other proteins where their interactions were mediated by both the PX and CC domains [49], [50]. The interaction of SEPT5 and SNX6 seems to be biologically relevant since several other proteins that have been described to interact with septins are also related to intracellular trafficking and/or exocytosis.

It is of interest to note that both SNX6 and CENP-E appear to interact with septins via a coiled coil domain. This may imply that under certain circumstances, acting individually or even within the context of certain heterofilaments, not all of the septin coiled coils are satisfied. Some combinations of septins may leave coiled coils available for interaction with non-septins partners (e.g. Fig. 4B).

In yeast it has been reported that septins recruit SDKs (septin dependent kinases) [53]–[55] indicating that phosphorylation of septins may be an important factor in the regulation of their activities. SDKs function as regulators of septin filament assembly and for the correct positioning and alignment of the microtubules during the budding process. Other kinases such as Hsl1 and Gin4 seem to be critical for the transition from G2 to M during the cell cycle [53], [56], [57] and their direct interaction with septins seems to be essential for their correct localization and activity. In our screens we identified two additional kinases that may represent candidates for additional points of regulation of septin activity. HIPK3 (Homeodomain interacting protein kinase 3) was found as an interactor for SEPT6 and PLK2 (Polo-like kinase 2) as an interactor of SEPT3 (Table S1). Although in both cases only a single clone was identified, the result may be significant. Based on the usually weak and transient interaction between kinases and their substrates, it is not expected that this type of interaction appear with considerable frequency in yeast two-hybrid screens. It is worth pointing out that PLK2 is like the functionally related SDKs mentioned above, associated with the regulation of the transition from G2 to M [58]. Should this interaction prove to be of physiological relevance our data suggest that aside the SDKs other kinases are involved in the functional regulation of septins during this part of the cell cycle.

In the case of the HIPK3 kinase, a possible biological link is less evident and there are only very few publications on the functions of this kinase currently available. It has been identified to be associated with the cytoplasmic domain of the FAS receptor, although its activity was not found to influence cell death directly [59].

Another interesting finding is the identification of the two proteins IFT27 (former name RabL4) and RalABP1 which interacted with SEPT3 and SEPT7 respectively. IFT27 is a Ras-like GTPase protein that contains the five consensus sequences needed for GTP-binding and GTPase activity. IFT27 can bind to GTP and may act at the end of the cytokinesis where Rab family members are involved in vesicle trafficking required to complete this process [60]. Since septins are also involved in vesicle trafficking and cytokinesis, the interaction with IFT27 may be biologically relevant and should be further tested experimentally. RalABP1 is a member of the Ras GTPase superfamily and the latter is GTPase-activator protein, involved in the regulation of endocytosis during interphase. Since the discovery that septins bind and hydrolyze GTP, although at a rather slow rate, it has been speculated that other interacting proteins may act as GAPs (GTPase activating proteins), that through promoting GTP to GDP hydrolysis may regulate septin function. This could alter the septins conformation and may influence its interaction with other proteins, including their assembly into septin filaments. RalABP1 has been already reported to act as a GAP on other GTPases and RabL4 could be another candidate for a septin GTPase regulatory protein. The GAP Rho has been previously shown to act on the mammalian SEPT9b [35].

Finally, we also found two interesting non-septin interactors of the SEPT9 N-terminal region: filamin A (FLNA) and SH3-domain kinase binding protein 1 (SH3KBP1). The former one is directly involved in actin cytoskeleton organization [61] and the interaction between FLNA and SEPT9 could represent a novel physical and functional connection between septin and actin filaments. SH3KBP1, the second interactor, is an adaptor protein involved in many processes, from cytoskeleton remodeling and vesicle-mediated transport to signal transduction and cell death [12], [62]–[65]. Recently, many proteins involved in cytoskeleton and membrane processes, including septins, were found to interact with SH3KBP1 by mass spectrometry analysis [66]. Our results confirm these previous studies since the N-terminal domain of SEPT9 picked up SH3KBP1 in the two-hybrid assay. In addition, an interactor of septins, cytoskeleton components and plasma membranes called anillin [67] was found to interact with SH3KBP1 [66]. Since anillin and septins interact directly [17] and both interact with SH3KBP1, our findings suggest that the septins-SH3KBP1 interaction could be involved in cytokinesis and plasma membrane processes, such as vesicle trafficking. Although our findings provide possible clues for better understanding septin filament assembly and regulation, further experimental studies are clearly essential.

### Prey regions involved in Protein-Protein Interactions

After DNA sequencing of the interacting prey plasmids and analysis of their sequences we found that a large fraction of the clones encode full-length or almost full length proteins. As expected however many clones also encode only protein fragments that may contain specific protein domains.

In case of the septin preys found to interact with septin baits it was especially obvious that the majority of clones encoded full length proteins (Table S1, e.g, SEPT3: interacted with SEPT6(4-427), SEPT11(7-429). Most interestingly, even if the prey septins were not full length proteins, they almost always encoded for the central GTPase domain, for example SEPT6, which interacted with SEPT9(129-568), SEPT5(39-369) and SEPT7(65-437), and SEPT4(101-478). This clearly confirms the known fact that the central GTPase domain is important for mediating septin-septin interactions [18]. Furthermore, this finding indicated that the identified new septin-septin interactions may indeed represent physiological relevant combinations. For the other, non-septin preys, we were able to identify the following overall trend: small-size prey proteins tend to be present rather completely, while for the larger proteins, often only a restricted interacting region could be identified. This may imply that for larger proteins, only specific domains or modules are responsible for septin recognition.

The first crystal structure of a septin complex [18] provided considerable food for thought with respect to the way in which this apparently redundant family of proteins can potentially form a myriad of different filaments. Here we provide the first large scale yeast two-hybrid study which addresses this question. We have provided a large body of experimental evidence which for the most part corroborates the speculation made by Kinoshita [6] that septins are substitutable within their given groups. New potential non-septin partners have also been described. What controls assembly and the physiological requirement for such potential diversity are questions which badly need addressing.

## Materials and Methods

### Plasmid construction

Oligonucleotides were designed to amplify and sub-clone the cDNAs encoding the amino acid sequences of the human Septins 2–10 studied here. Full length SEPT1 was picked up as a prey in a two hybrid screen with SEPT6. Its cDNA was subsequently sub-cloned in the pBTM116 vector to perform a screen with SEPT1 as bait. In all cases full length cDNAs were amplified, except for SEPT9, where in addition to the full length protein we also used a construct that spans the N-terminal region alone (amino acids 1- 269). A second exception was that of the full length version of SEPT4, which showed auto-activation of the reporter genes. In this case we employed for the screen a construct which lacked the N-terminal domain (aa 124-478) and no longer resulted in auto-activation. All septin cDNAs were isolated from a human fetal brain cDNA library (Clontech). These were cloned in frame with lexA into the poly linker of the bait vector pBTM116 which had the ampicilin resistance marker changed to a kanamycin resistance marker to facilitate recovery of the prey plasmid from the co-transformed bait-plasmid pACT2, contains an Amp resistance marker. Furthermore, the modified plasmid contained some additional restriction enzyme sites in the cloning site to facilitate cloning. A total of 11 baits cDNAs were successfully cloned. These include: hSEPT1 (NM_052838), hSEPT2 (NM_004404), hSEPT3 (NM_019106), hSEPT4 (lacking the N-terminal domain and hence corresponding to amino acid residues 124–478) (NM_080416), hSEPT5 (NM_002688), hSEPT6 (NM_015129), hSEPT7 (NM_001788), hSEPT8 (NM_001098811.1), hSEPT9 (NM_006640) transcript variant 3, protein: isoform c, hSEPT9 N-terminal region (amino acid residues 1-269 transcript variant 3, protein: isoform c), and hSEPT10 (NM144710). These are no new cell lines but only cDNA clones obtained by in vitro experiments as described above.

### Basic yeast procedures and two hybrid screen

The yeast two-hybrid screens [68] of two different cDNA libraries were screened for all 11 bait proteins; a human fetal brain library and a human leukocyte library (both from Clontech). We used the yeast strain L40 (*trp1-901*, *his3Δ200*, *leu2-3*, *ade2 LYS2::(lexAop)4-HIS3 URA3::(lexAop)8-lac GAL4*) and the baits described above fused to the bacterial LexA protein in the slightly modified vector pBTM116 [69]. Since SEPT4 auto-activated the yeast reporter genes, a construct spanning amino acids 124-478 was used, which showed no auto-activation. This construct contains the GTPase and C-terminal domains of SEPT4, but lacks the relatively large N-terminal domain.

Yeast cells were transformed according to procedures provided by Clontech. The autonomous activation test for HIS3 was performed in minimal medium plates in the absence of tryptophan and histidine but containing varying concentrations of 3-AT (3-amino-1,2,4-triazole). None of the septin bait constructs, except SEPT4, showed auto-activation.

For the library scale screens the competent L40 yeast cells were first transfected with the bait construct as described previously [70]. The recombinant cells were then, in a second round of growth, transfected with the library plasmid. For the interaction screen these double transfected cells were then plated on minimal medium plates in the absence of tryptophan, leucine, and histidine and containing 5 mM 3-AT, to suppress non specific background growth. At least 1 million co-transfectants were plated and analyzed in total for each bait septin. Typically this meant that at least 20 plates of 15 cm diameter were screened for each of the two libraries. The number of clones growing varied greatly from septin to septin bait, ranging from few clones for SEPT10 to several thousand clones in case of SEPT6. Recombinant pACT2 plasmids of growing colonies were isolated and subsequently transformed in *E. coli* for plasmid amplification and isolation. Prey plasmid DNA was extracted and sequenced with an automatic DNA sequencer (Model 16-capillary 3130xl Genetic Analyzer, Applied Biosystems). The corresponding Accession numbers of the DNA sequences identified are given in the Supplementary Table S1. As no new sequences have been obtained no new sequence data have been deposited in the GenBank.

When only a relatively small number of colonies grew (<100-), plasmid DNA was extracted and sequenced from all such colonies. In cases where a relatively large number of colonies were obtained (>1000), plasmid was extracted and sequenced for at least 200 colonies (SEPT6 and SEPT8). All plasmids were submitted to confirmation assays in the yeast (see next paragraph) and only those which proved to be positive were considered for subsequent analyses and are presented here (see next paragraph). In some cases up to 50% of the initially sequenced clones did not give positive results in the confirmation assay and all of these were discarded from further analysis. All clones shown in the supplementary tables 1–10 have been confirmed.

### Yeast interaction confirmation assay

Extracted plasmids from positive clones during the initial screening were used to transform L40 yeast cells previously transformed with the appropriate bait. The interactions were confirmed in yeast cells using the β-galactosidase assay. The control of assay was performed with an empty bait vector (pBTM116-lexA alone). Clones that did not confirm the growth and blue color production when co-transformed with their bait septins were discarded from further analysis (false positives). Any prey plasmids that had alone (in absence of the specific bait vector and presence of “empty” bait vector only) the capacity to promote growth or the production of blue color was also discarded (false positive). All clones reported in the Table S1 have been confirmed.

### β-Galactosidase assay for the confirmation of interactions

For confirmation of the potential interaction between the septin bait and the fished prey clones in a one-to-one fashion, β-Galactosidase activity in yeast cells was determined using the filter assay method. Yeast transformants (Leu+, Trp+, His+) grown on minimal medium were transferred onto filter papers. The paper disks were incubated for 3 min in liquid nitrogen, thawed and soaked with Z buffer (60 mM Na_2_HPO_4_, 40 mM NaH_2_PO_4_, 10 mM MgCl_2_, 50 mM 2-mercaptoethanol, pH 7.0) containing 1 mg.mL^−1^ 5-bromo-4-chloro-3-indolyl-β-D-galactoside (X-Gal). After incubation at 37°C for 30 min to 1 h the formation of a blue color was evaluated. Only clones that had an unambiguous blue color were considered true positives. These interactions were considered to be confirmed and the corresponding clones were included in the data analysis presented in the tables and figures of this report. Colonies that remained white or faintly blue were excluded from further analyses.

### Cloning, protein expression and purification for confirmatory interaction assays

For expression in *E. coli* of either the bait proteins or the corresponding preys identified above, the encoding cDNAs were sub-cloned into the expression vectors pET28 and/or pGEX as described [70]. Orientation and correctness of DNA sequences were confirmed by DNA sequencing. The recombinant proteins were either expressed in fusion with GST or 6xHis tags according to standard protocols [71]. Subsequently, the fusion proteins were purified on glutathione-Uniflow resin (Clontech) or HiTrap chelating resin (GE Healthcare) as described [72]. *In vitro* binding assays/pull down assays were performed as described previously [73].

### Statistical analysis

In the case of the septin bait's interactions with other prey septins (Fig. 2, 3) we performed group wise statistical analysis using a Fischeŕs exact test for counts employing the free software R, version 2.11.1 [74]. First, septins were grouped into four groups, according to their sequence similarity: group 2 (septins 1,2,4,5), group 6 (septins 6,8,10,11), group 3/9 (septins 3 and 9), “group” 7 (only septin 7). Then the number of interacting clones was summed for each member inside the same group (see raw data plotted in Fig. 2 and also Table S1) and tested for a random distribution. Subsequently, we compared groups in a one-to-one and reciprocal fashion in a similar manner. Finally, an analysis was performed for the occurrence of interactions among septins of the same group. Please refer to the supplementary material for detailed results of the statistical analysis.

## Supporting Information

Table S1 Characteristics of interacting proteins for septins 1 to septin 10 as predicted from the clones retrieved in the yeast two-hybrid system screenings. The Tables appear in sequence of the septin protein used as bait in the screen. HFB: Human fetal brain cDNA library screened, LEU: human leukocyte cDNA library screened. The number of clones obtained is indicated as a total as well as the numbers for the leukocyte and human fetal brain library separately. The gene accession number, main assigned protein function, specific present protein domains and references are also given. Septins are listed first followed by non-septins, in both cases in order of decreasing frequency (i.e., the number of identified clones, independent of being either identical or not. See table for discrimination if available). Results of statistical analyses are given after the tables.
(0.39 MB DOC)

Figure S1 A protein interaction network of the human septins 1-10. The network consists of a total of proteins (colored nodes, including the septin baits and its interacting partners identified in the yeast two-hybrid screens) and the interactions connecting them (grey links). The nodes are colored based on the GO biological process as indicated in the legend. The network was generated using the Osprey 1.2.0. software (http://biodata.mshri.on.ca/osprey/). The proteins that interacted with septin are involved in Carbohydrate Metabolism, Cell Cycle, Cell Organization and Biogenesis, DNA Damage Response, DNA metabolism, DNA Repair, Metabolism, Protein amino acid phosphorylation, Protein biosynthesis, Protein transport, RNA Localization, RNA processing, Signal transduction, Transcription, Transport.
(0.11 MB DOC)
