# Supplementary material for: A Draft of the Human Septin Interactome
Source: PLoS One. 2010 Nov 2;5(11):e13799. doi: 10.1371/journal.pone.0013799 (PMC2970546; doi:10.1371/journal.pone.0013799)
Supplement: Table S1 — Characteristics of interacting proteins for septins 1 to septin 10 as predicted from the clones retrieved in the yeast two-hybrid system screenings. The Tables appear in sequence of the septin protein used as bait in the screen. HFB: Human fetal brain cDNA library screened, LEU: human leukocyte cDNA library screened. The number of clones obtained is indicated as a total as well as the numbers for the leukocyte and human fetal brain library separately. The gene accession number, main assigned protein function, specific present protein domains and references are also given. Septins are listed first followed by non-septins, in both cases in order of decreasing frequency (i.e., the number of identified clones, independent of being either identical or not. See table for discrimination if available). Results of statistical analyses are given after the tables. (0.39 MB DOC) [file pone.0013799.s001.doc]

**Septin 1 bait**

| Protein name/ abreviation | Number of clones  **Total**/HFB/ LEU | Coded protein residues | Acession number | Function | Protein domains (in retrieved regions) | Reference |
| --- | --- | --- | --- | --- | --- | --- |
| Septin 1  (SEPT1) | **03**/ - / 03 | (372)  1-372 | [NM_052838.2](http://www.ncbi.nlm.nih.gov/nuccore/NM_052838.2) | Cell division, cell cycle | Full length | Mori, et al, 1996 |
| Septin 2 (SEPT2) | **02** /01 / 01 | (367)  1-367 | BAA09928 | Cell division, cell cycle, cytokinesis | Full length | Kinoshita, *et al*, 1997 |
| Septin 6 (SEPT6) | **14** / 01 / 13 | (491)  1-491 | EAW89857 | Cell division, cell cycle | Full length | Ono, *et al*, 2002 |
| Septin9 (SEPT9) | **15** / 0 / 15 | (422)  1-422  32-422  77-422  126-422  69-422 | NM_001113492 | Cell cycle, cell division | Full length  GC | Osaka, *et al,* 1999 |
| Septin 11 (SEPT11) | **03** / 03 / - | (429)  1-429  32-429 | NM_018243 | Cell cycle, cell division | Full length  GC | Hanai, *et al*, 2004 |
| Septin 4 (SEPT4) | **01** / - / 01 | (459)  78-459 | NM_080416 | Cell cycle, cytokinesis, regulation of apoptosis | GC | Xie, *et al*, 1999 |
| Septin 5 (SEPT5) | **01** / - / 01 | (369)  18-369 | NM_002688 | Cell cycle, cytokinesis, regulation of exocytosis, synaptic vesicle targetin | GC | Yagi, *et al*, 1998 |
| Ubiquitin-conjugating enzyme E2I (UBE2I) | **03**/ 03/ - | (158)  1-158  7-158 | NM_003345 | [Cell cycle](http://www.ncbi.nlm.nih.gov/entrez/utils/fref.fcgi?http://amigo.geneontology.org/cgi-bin/amigo/go.cgi?view=details&depth=1&query=7049), Cell division, regulation of protein metabolic process, [mitosis](http://www.ncbi.nlm.nih.gov/entrez/utils/fref.fcgi?http://amigo.geneontology.org/cgi-bin/amigo/go.cgi?view=details&depth=1&query=7067), ubiquitin cycle | Full lenght | Watanabe, et al, 1996 |
| spindle and kinetochore-associated protein 2 isoform 1-SKA1 | **01** / - / 01 | (121)  1 - 121 | NM_182620 | maintenance of the metaphase plate and/or spindle checkpoint silencing | Full lenght | Hanisch *et al*, 2006 |
| centriole associated protein CEP110 | **02** / - / 02 | (994)  660-994  567-994 | AF083322 | centrosome maturation. | c-terminal domain | Young *et a,l* 2002 |

**Septin 2 bait**

| Protein name/ abreviation | Number of clones  **Total**/HFB/ LEU | Coded protein residues | Acession number | Function | Reference |
| --- | --- | --- | --- | --- | --- |
| Septin 4 (SEPT4) | **01 / - /** 01 |  | EAW94449.1 | Apoptosis, cell cycle, cytokinesis | Xie, *et al*, 1999 |
| Septin 6 (SEPT6) | **05** / 03 / 02 | 491/26-340 | EAW89853.1  EAW89857.1 | Cell cycle, cell division, cytokinesis, heterooligomerization | Kinoshita, *et al*, 2000 |
| Ankyrin repeat and zinc finger domain containing 1 (ANKZF1) | **01 / - /** 01 | (726) 41/728 | EAW70708 | Metal ion binding, zinc ion binding | Venter, *et al*, 2001 |
| Dynactin 2 (DCTN2) | **01 / - /** 01 | (406) 157/314 | [EAW97028](http://www.ncbi.nlm.nih.gov/entrez/viewer.fcgi?db=protein&val=119617434) | Cell proliferation, mitosis, mitotic spindle organization | Venter, *et al*, 2001 |
| coiled-coil domain containing 45 (CCDC45) | **01 / - /** 01 | (821) 194-211 | BAF83276.1 | Protein binding | Wakamatsu, *et al* |
| Proprotein convertase subtilisin/kexin type 1 inhibitor-  PC-S/K-I | **01 /** 01/ **-** | 260/180-247 | NP_037403.1 | Members of the subtilisin-like proprotein convertase  family process latent precursor proteins into their biologically  active products. The protein encoded by this gene appears to  function as an endogenous inhibitor of proprotein convertase  subtilisin/kexin type 1 | Wada et al., 2004 |
| MAP3K12-binding inhibitory protein 1 (MAPK upstream kinase-binding inhibitory protein) (MUK-binding nhibitory protein) | **01 /** 01/ **-** | 344/149-344 | Q9NS73.2 | Inhibits the MAP3K12 activity to induce the activation  of the JNK/SAPK pathway | Fukuyama et al., 2000 |
| Ribosomal protein S6 kinase-like 1, isoform CRA_d | **01 /** 01/ **-** | 389/367-389 | EAW81206.1 | Phosphotransferases of the serine or threonine-specific  kinase subfamily | Venter, *et al*, 2001 |
| Ubiquitin-conjugating enzyme E2I (UBC9 homolog, yeast) | **01 /** 01/ **-** | 188/5-188 | AAH51289.3 | Ubiquitin-conjugating enzyme E2, catalytic (UBCc)  domain. This is part of the ubiquitin-mediated protein  degradation pathway | Watanabe, et al, 1996 |

**Septin 3 bait**

| Protein name/ abreviation | Number of clones  **Total**/HFB/ LEU | Coded protein residues | Acession number | Function | Protein domains (in retrieved regions) | Reference |
| --- | --- | --- | --- | --- | --- | --- |
| Septin 6  (SEPT6) | **06** / 06 / **-** | (427)  4-427 | [NM_145799.3](http://www.ncbi.nlm.nih.gov/nuccore/NM_145799.3) | cytokinesis and cell cycle control | Full length  NGC | Kinoshita, *et al*, 2000 |
| Septin 11  (SEPT11) | 05 / 05 / **-** | (429)  7-429 | [NM_018243.2](http://www.ncbi.nlm.nih.gov/nuccore/NM_018243.2) | cytokinesis and cell cycle control | Full length  NGC | Hanai et al., 2004 |
| Actin beta  (ACTB) | **01** / 01 / **-** | (375)  219-375 | [NM_001101.3](http://www.ncbi.nlm.nih.gov/nuccore/NM_001101.3) | Cell motility, structure and integrity | Partial actin domain | Vandekerckhove, et al, 1978 |
| ATP-binding cassette, sub-family B (MDR/TAP), member 10 (ABCB10) | **01** / 01 / **-** | (738)  195-738 | NM_012089 | [transmembrane transport](http://www.ncbi.nlm.nih.gov/entrez/utils/fref.fcgi?http://amigo.geneontology.org/cgi-bin/amigo/go.cgi?view=details&depth=1&query=55085), [transport](http://www.ncbi.nlm.nih.gov/entrez/utils/fref.fcgi?http://amigo.geneontology.org/cgi-bin/amigo/go.cgi?view=details&depth=1&query=6810) | (171) ABC membrane and AAA domains | Allikmets, et al, 1995 |
| Caspase 8 associated protein 2 (CASP8AP2) | **05** / 05 / - | (1982)  987-1982 | NM_012115 | [Activation of caspase activity](http://www.ncbi.nlm.nih.gov/entrez/utils/fref.fcgi?http://amigo.geneontology.org/cgi-bin/amigo/go.cgi?view=details&depth=1&query=6919), apoptosis, [cell cycle](http://www.ncbi.nlm.nih.gov/entrez/utils/fref.fcgi?http://amigo.geneontology.org/cgi-bin/amigo/go.cgi?view=details&depth=1&query=7049), [regulation of transcription](http://www.ncbi.nlm.nih.gov/entrez/utils/fref.fcgi?http://amigo.geneontology.org/cgi-bin/amigo/go.cgi?view=details&depth=1&query=45449) | SANT domain | Imai, et al, 1999 |
| Eukaryotic translation initiation factor 4A (EIF4A) | **01** / 01 / - | (406)  103-406 | NM_001416 | [Translation](http://www.ncbi.nlm.nih.gov/entrez/utils/fref.fcgi?http://amigo.geneontology.org/cgi-bin/amigo/go.cgi?view=details&depth=1&query=6412) | Part of DEADc domain and HELICc domain | Reddy, et al, 1988 |
| exosome component 9 (EXOSC9) | 01 / 01 / - | (439)  138-439 | NM_005033 | [RNA processing](http://www.ncbi.nlm.nih.gov/entrez/utils/fref.fcgi?http://amigo.geneontology.org/cgi-bin/amigo/go.cgi?view=details&depth=1&query=6396), [immune response](http://www.ncbi.nlm.nih.gov/entrez/utils/fref.fcgi?http://amigo.geneontology.org/cgi-bin/amigo/go.cgi?view=details&depth=1&query=6955) | RNAse PH C domain | Alderuccio, et al, 1991 |
| GABA(A) receptor-associated protein-like 2 (GABARAPL2) | 01 / 01 / - | (117)  1-117 | NM_007285 | [Regulation of ATPase activity](http://www.ncbi.nlm.nih.gov/entrez/utils/fref.fcgi?http://amigo.geneontology.org/cgi-bin/amigo/go.cgi?view=details&depth=1&query=32781), [transport](http://www.ncbi.nlm.nih.gov/entrez/utils/fref.fcgi?http://amigo.geneontology.org/cgi-bin/amigo/go.cgi?view=details&depth=1&query=15031) | GABARAP domain | Sagiv, et al, 2000 |
| Heterogeneous nuclear ribonucleoprotein H3 (HNRNPH3) | 01 / 01 / - | (346)  84-346 | NM_012207 | [RNA processing](http://www.ncbi.nlm.nih.gov/entrez/utils/fref.fcgi?http://amigo.geneontology.org/cgi-bin/amigo/go.cgi?view=details&depth=1&query=6396), [RNA splicing](http://www.ncbi.nlm.nih.gov/entrez/utils/fref.fcgi?http://amigo.geneontology.org/cgi-bin/amigo/go.cgi?view=details&depth=1&query=8380) | RRM domain | Rasmussen, et al, 1992 |
| Myosin IB (MYO1B) | 01 / 01 / - | (1078)  701-1078 | NM_012223 | Involved in the traffic along the endocytic pathway | IQ and Myosin TH1 domains | Ruppert, et al, 1993 |
| Peroxiredoxin 2 (PRDX2) | 01 / 01 / - | (198)  1-198 | NM_005809 | [Anti-apoptosis](http://www.ncbi.nlm.nih.gov/entrez/utils/fref.fcgi?http://amigo.geneontology.org/cgi-bin/amigo/go.cgi?view=details&depth=1&query=6916), [activation of MAPK activity](http://www.ncbi.nlm.nih.gov/entrez/utils/fref.fcgi?http://amigo.geneontology.org/cgi-bin/amigo/go.cgi?view=details&depth=1&query=187), [cell redox homeostasis](http://www.ncbi.nlm.nih.gov/entrez/utils/fref.fcgi?http://amigo.geneontology.org/cgi-bin/amigo/go.cgi?view=details&depth=1&query=45454), [T cell proliferation](http://www.ncbi.nlm.nih.gov/entrez/utils/fref.fcgi?http://amigo.geneontology.org/cgi-bin/amigo/go.cgi?view=details&depth=1&query=42098) | AhpC-TSA and 1-cysPrx C domains | Rasmussen, et al, 1992 |
| polo-like kinase 2 (PLK2) | 01 / 01 / - | (685)  440-685 | NM_006622 | [Mitotic cell cycle](http://www.ncbi.nlm.nih.gov/entrez/utils/fref.fcgi?http://amigo.geneontology.org/cgi-bin/amigo/go.cgi?view=details&depth=1&query=278), [phosphorylation](http://www.ncbi.nlm.nih.gov/entrez/utils/fref.fcgi?http://amigo.geneontology.org/cgi-bin/amigo/go.cgi?view=details&depth=1&query=6468) | POLO box domain | Kauselmann, et al, 1999 |
| Protein inhibitor of activated STAT3 (PIAS3) | 04 / 04 / - | (628)  110-628  336-628 | NM_006099 | [Regulation of protein sumoylation](http://www.ncbi.nlm.nih.gov/entrez/utils/fref.fcgi?http://amigo.geneontology.org/cgi-bin/amigo/go.cgi?view=details&depth=1&query=33235), [regulation of transcription](http://www.ncbi.nlm.nih.gov/entrez/utils/fref.fcgi?http://amigo.geneontology.org/cgi-bin/amigo/go.cgi?view=details&depth=1&query=45449), [response to hormone stimulus](http://www.ncbi.nlm.nih.gov/entrez/utils/fref.fcgi?http://amigo.geneontology.org/cgi-bin/amigo/go.cgi?view=details&depth=1&query=9725) | zf-MIZ domain | Chung, et al, 1997 |
| Intraflagellar transport 27 (IFT27) | **01** / 01 / - | (185)  22-185 | NM_006860 | [Rab-like](http://www.ncbi.nlm.nih.gov/entrez/utils/fref.fcgi?http://amigo.geneontology.org/cgi-bin/amigo/go.cgi?view=details&depth=1&query=7264) small G protein, cell-cycle control | Small GTPase domain | Qin, et al, 2007 |
| Ribosomal protein L14 (RPL14) | 01 / 01 / - | (215)  40-215 | NM_003973 | [rRNA processing](http://www.ncbi.nlm.nih.gov/entrez/utils/fref.fcgi?http://amigo.geneontology.org/cgi-bin/amigo/go.cgi?view=details&depth=1&query=6364), [translation](http://www.ncbi.nlm.nih.gov/entrez/utils/fref.fcgi?http://amigo.geneontology.org/cgi-bin/amigo/go.cgi?view=details&depth=1&query=6412) | Ribosomal L14e domain | Li, et al, 1993 |
| Ribosomal protein S24 (RPS24) | **01** / 01 / **-** | (130)  1-130 | NM_033022 | [rRNA processing](http://www.ncbi.nlm.nih.gov/entrez/utils/fref.fcgi?http://amigo.geneontology.org/cgi-bin/amigo/go.cgi?view=details&depth=1&query=6364), [translation](http://www.ncbi.nlm.nih.gov/entrez/utils/fref.fcgi?http://amigo.geneontology.org/cgi-bin/amigo/go.cgi?view=details&depth=1&query=6412) | Ribosomal S24e domain | Brown, et al, 1990 |
| Stathmin-like 2 (STMN2) | 01 / 01 / - | (179)  1-179 | NM_007029 | [Intracellular signaling cascade](http://www.ncbi.nlm.nih.gov/entrez/utils/fref.fcgi?http://amigo.geneontology.org/cgi-bin/amigo/go.cgi?view=details&depth=1&query=7242), [neuron differentiation](http://www.ncbi.nlm.nih.gov/entrez/utils/fref.fcgi?http://amigo.geneontology.org/cgi-bin/amigo/go.cgi?view=details&depth=1&query=30182) | Stathmin domain | Okazaki, et al, 1995 |
| SMT3 supressor of mif two 3 homolog 1 (SUMO1) | 01 / 01 / - | (101)  1-101 | NM_003352 | Protein sumoylation, [regulation of protein localization](http://www.ncbi.nlm.nih.gov/entrez/utils/fref.fcgi?http://amigo.geneontology.org/cgi-bin/amigo/go.cgi?view=details&depth=1&query=32880) | UBQ domain | Shen, et al, 1996 |
| Transmembrane protein 93 (TMEM93) | **01** / 01 / - | (110)  10-110 | NM_031298 | [integral to membrane](http://www.ncbi.nlm.nih.gov/entrez/utils/fref.fcgi?http://amigo.geneontology.org/cgi-bin/amigo/go.cgi?view=details&depth=1&query=16021), non-defined | Rab5ip superfamily domain | Oh, et al, 2005 |
| Ubiquitin-conjugating enzyme E2I (UBE2I) | **19** / 19 / - | (158)  1-158, 7-158, 39-158 | NM_003345 | Cell division, regulation of protein metabolic process, ubiquitin cycle | UBCc domain | Watanabe, et al, 1996 |
| Thymine-DNA glycosylase-TDG | **02** / 02 / - | (410)  24-410, 89-158 | NM_003211 | DNA repair, DNA demethylase, gene regulation | UDG domain | Cortázar et al., 2007 |

( ⇑ septin 3 bait continued ⇑ )

**Septin 4 (amino acids 124-478) bait**

| Protein name/ abreviation | Number of clones  **Total**/HFB/ LEU | Coded protein residues | Acession number | Function | Reference |
| --- | --- | --- | --- | --- | --- |
| Septin 6 (SEPT6) | **2**/ 2 / - | 4-251 | NM_145802 | Cell cycle, cell division, cytokinesis, heterooligomerization | Ono et al., 2003 |
| Septin 8 (SEPT8) | **1** / 01 / - | 51-164 | NM_015146 | Cell cycle, cell division | Bläser et al., 2003 |
| Septin 10 (SEPT10) | **03** / 03 / - | 1-103 | NM_144710 | Cell cycle, cell division, | Sui et al., 2003 |
| Septin 11 (SEPT11) | **07** / 07 / - | 7-289 | NM_018243 | Cell cycle, cell division | Hanai et al., 2004 |
| CASC3 | **01** / 01 / - | 703/ 592-641 | NM_007359 | RNA splicing, regulation of translation, transport | Tomasetto, *et al*, 1995 |
| Ubiquitin-conjugating enzyme E2I (UBE2I) | **08**/ 08/ - | 158/ 1-158 | NM_003345 | Cell division, regulation of protein metabolic process, ubiquitin cycle | Watanabe et al., 1996 |
| VEGFR-1 | **03** / 03 / - | 1338/ 1103-1189 | NM_002019 | Cell differentiat., positive regulation cell migration, Receptor Tyr kinase | Matsushime, *et al*, 1987 |

**Septin 5 bait**

| Protein name/ abreviation | Number of clones  **Total**/HFB/ LEU | Coded protein residues | Acession number | Function | Protein domains (in retrieved regions) | Reference |
| --- | --- | --- | --- | --- | --- | --- |
| Septin 2 (SEPT2) | **01** / - / 01 | (367)  1-367 | BAA09928 | Cell division, cell cycle, cytokinesis | Full length | Kinoshita, *et al*, 1997 |
| Septin 5 (SEPT5) | **01** / 01 / - | (369)  1-369 | NM_002688 | Cell cycle, cytokinesis, regulation of exocytosis, synaptic vesicle targetin | Full length | Yagi, *et al*, 1998 |
| Septin 6 (SEPT6) | **08** / 01 / 07 | (491)  1-491 | EAW89857 | Cell division, cell cycle | Full length | Ono, *et al*, 2002 |
| Septin 8 (SEPT8) | **06** / 06 / - | (429)  1-429 | NM_015146 | Cell cycle, cell division, platelet granular secretion | NGC | Bläser, *et al*, 2003 |
| Septin 11 (SEPT11) | **03** / 03 / - | (429)  1-429 | NM_018243 | Cell cycle, cell division | Full length | Hanai, *et al*, 2004 |
| sorting nexin 6 (SNX6) | **01** / - / 01 | (406)  307-406 | AAD27829 | intracellular  trafficking | C-terminal dimerisation domain | Parks, *et al*, 2001 |
| ubiquitin-conjugating enzyme E2I (UBE21) | **06** / 03 / 03 | (158)  1-158  7-158  8-158  9-158 | NM_003345 | [Cell cycle](http://www.ncbi.nlm.nih.gov/entrez/utils/fref.fcgi?http://amigo.geneontology.org/cgi-bin/amigo/go.cgi?view=details&depth=1&query=7049), Cell division, regulation of protein metabolic process, [mitosis](http://www.ncbi.nlm.nih.gov/entrez/utils/fref.fcgi?http://amigo.geneontology.org/cgi-bin/amigo/go.cgi?view=details&depth=1&query=7067), ubiquitin cycle | Full length | Watanabe, *et al*, 1996 |

**Septin 6 bait**

| Protein name/ abreviation | Number of clones  **Total**/HFB/ LEU | Coded protein residues | Acession number | Function | Reference |
| --- | --- | --- | --- | --- | --- |
| Septin 9 (SEPT9) | **96**/ 32 / 64 | (586)  129-568  169-568  222-568  263-568 | NM_006640 | Cell cycle, cell division, heterooligomerization | Osaka, *et al,* 1999 |
| Septin 5 (SEPT5) | **12** / 08 / 04 | (369)  1-369  17-369  39-369  30-369 | NM_002688 | Cell cycle, cytokinesis, regulation of exocytosis, synaptic vesicle targeting | Yagi, *et al*, 1994 |
| Septin 7 (SEPT7) | **09** / 05 / 04 | (436)  65-437  23-436 | NM_001788 | Cell cycle, cell division, cytokinesis, heterooligomerization | Nakatsuru, et al, 1994 |
| Septin 1 (SEPT1) | **08** / 0 / 08 | (367)  1-367 | NM_052838 | Cell cycle, cell division | Mori, et al, 1996 |
| Septin 4 (SEPT4) | **05** / 03 / 02 | (459)  1-230  101-478 | NM_080416 | Cell cycle, cytokinesis, regulation of apoptosis | Xie, *et al*, 1999 |
| Septin 2 (SEPT2) | **05** / 02 / 03 | (361)  1-361 | NM_004404 | Cell cycle, cell division, [mitosis](http://www.ncbi.nlm.nih.gov/entrez/utils/fref.fcgi?http://amigo.geneontology.org/cgi-bin/amigo/go.cgi?view=details&depth=1&query=7067), [regulation of protein localization](http://www.ncbi.nlm.nih.gov/entrez/utils/fref.fcgi?http://amigo.geneontology.org/cgi-bin/amigo/go.cgi?view=details&depth=1&query=32880) | Mori, *et al*, 1996 |
| Septin 3 (SEPT3) | **01** / 01 / 0 | (358)  1-210 | NM_145733 | Cell cycle, cytokinesis | Dunham, et al, 1999 |
| Ubiquitin-conjugating enzyme E2I (UBE2I) | **07**/ 07/ 0 | (158)  1-158  7-158 | NM_003345 | [Cell cycle](http://www.ncbi.nlm.nih.gov/entrez/utils/fref.fcgi?http://amigo.geneontology.org/cgi-bin/amigo/go.cgi?view=details&depth=1&query=7049), Cell division, regulation of protein metabolic process, [mitosis](http://www.ncbi.nlm.nih.gov/entrez/utils/fref.fcgi?http://amigo.geneontology.org/cgi-bin/amigo/go.cgi?view=details&depth=1&query=7067), ubiquitin cycle | Watanabe, et al, 1996 |
| Small ubiquitin-like modifier (SUMO) | **03**/ 03/ 0 | (101)  1-101 | NM_003352 | [DNA repair](http://www.ncbi.nlm.nih.gov/entrez/utils/fref.fcgi?http://amigo.geneontology.org/cgi-bin/amigo/go.cgi?view=details&depth=1&query=6281), Protein sumoylation, [regulation of protein localization](http://www.ncbi.nlm.nih.gov/entrez/utils/fref.fcgi?http://amigo.geneontology.org/cgi-bin/amigo/go.cgi?view=details&depth=1&query=32880) | Shen, *et al*, 1996 |
| CASP8 associated protein 2 (CASP8AP2) | **02**/ 02/ 0 | (1986)  1368-1710 | NM_012115 | Apoptosis, caspase activation, cell cycle, [transcription](http://www.ncbi.nlm.nih.gov/entrez/utils/fref.fcgi?http://amigo.geneontology.org/cgi-bin/amigo/go.cgi?view=details&depth=1&query=45449) | Imai, *et al*, 1999 |
| Protein inhibitor of activated STAT (PIAS3) | **02**/ 02/ 0 | (628)  110-400 | NM_006099 | DNA binding,  [regulation of protein sumoylation](http://www.ncbi.nlm.nih.gov/entrez/utils/fref.fcgi?http://amigo.geneontology.org/cgi-bin/amigo/go.cgi?view=details&depth=1&query=33235), transcription | Chung, et al, 1997 |
| Topoisomerase I binding, arginine/serine-rich (TOPORS) | **02**/ 02/ 0 | (1045)  590-900 | NM_005802 | [Apoptosis](http://www.ncbi.nlm.nih.gov/entrez/utils/fref.fcgi?http://amigo.geneontology.org/cgi-bin/amigo/go.cgi?view=details&depth=1&query=6915), Response to DNA damage stimulus, transcription, ubiquitin cycle | Haluska, et al, 1999 |
| Actin-related protein 2 (ACTR2) | **01**/ 0/ 01 | (399)  224-247 | NM_005722 | Cell motility | Welch, et al, 1997 |
| HIPK3 | **01**/ 0/ 01 | (1215)  865-1050 | NM_001048200 | Anti-apoptosis, apoptosis, negative regulation of JNK activity, peptidyl-S/T phosphorylation | Begley, et al, 1997 |
| Malate dehydrogenase 1 (MDH1) | **01**/ 01/ 0 | (334)  1-290 | NM_005917 | NAD metabolic process, glycolysis, cellular carbohydrate metabolic process | Larson et al., 1982 |

**Septin 7 bait**

| Protein name/ abreviation | Number of clones  **Total**/HFB/ LEU | Coded protein residues | Acession number | Function | Protein domains (in retrieved regions) | Reference |
| --- | --- | --- | --- | --- | --- | --- |
| Septin 1  (SEPT1) | **01** / - / 01 | (372)  12-218 | [NM_052838.2](http://www.ncbi.nlm.nih.gov/nuccore/NM_052838.2) | Cell division, cell cycle | NG | Mori, et al, 1996 |
| Septin 4  (SEPT4) | **03** / 03 / - | (478)  53-459  42-459 | NM_080416 | Cell cycle, cytokinesis, regulation of apoptosis | GC | Xie, *et al*, 1999 |
| Septin 6  (SEPT6) | **11** / 01 / 10 | (491)  90- 491  1-491, 4-491, 3-491 | EAW89857 | Cell division, cell cycle | Full length  GC | Ono, *et al*, 2002 |
| Septin 9  (SEPT9) | **34** / 01 / 33 | (422)  1 - 422  117-422, 77-422  126-422, 32-422  95-422, 69-422 | NM_001113492 | Cell cycle, cell division | Full length  GC | Osaka, *et al,* 1999 |
| Septin 10  (SEPT10) | **01** / 01 / - | (454)  1-454 | [NM_144710.2](http://www.ncbi.nlm.nih.gov/nuccore/NM_144710.2) | Cell cycle, cell division | Full length | Sui, *et al*, 2003 |
| Septin 11  (SEPT11) | **01** / 01 / - | (429)  219 - 429 | NM_018243 | Cell cycle, cell division | GC | Hanai, *et al*, 2004 |
| Ankirin repeat domain 12 (ANKRD12) | **01** / 01 / - | (2062)  379-642 | [NM_015208.3](http://www.ncbi.nlm.nih.gov/nuccore/NM_015208.3) | Nuclear protein  Unknown function | protein-protein interaction domain | Zhang, *et al*, 2004 |
| ralA binding protein 1  (RALBP1) | **02** / - / 02 | (655)  511-655 | [NM_006788.3](http://www.ncbi.nlm.nih.gov/nuccore/NM_006788.3) | [Regulation of GTPase activity](http://www.ncbi.nlm.nih.gov/entrez/utils/fref.fcgi?http://amigo.geneontology.org/cgi-bin/amigo/go.cgi?view=details&depth=1&query=43087), [transport](http://www.ncbi.nlm.nih.gov/entrez/utils/fref.fcgi?http://amigo.geneontology.org/cgi-bin/amigo/go.cgi?view=details&depth=1&query=6810) | C-terminal domain | Jullien-Flores, *et al*, 1995 |
| zinc finger protein 451 (ZNF451) | **01** / - / 01 | (559)  1-559 | CAH71222 | [regulation of transcription](http://www.ncbi.nlm.nih.gov/entrez/utils/fref.fcgi?http://amigo.geneontology.org/cgi-bin/amigo/go.cgi?view=details&depth=1&query=45449) | Full length | Karvonen, *et al*, 2008 |

**Septin 8 bait**

| Protein name/ abreviation | Number of clones  **Total**/HFB/LEU | Coded protein residues | Acession number | Function | Reference |
| --- | --- | --- | --- | --- | --- |
| Septin 9 (SEPT9) | **49** / 11 / 38 | (586)  13-568, 15-500, 32-568, 63-568, 97-500, 234-568, 240-568, 249-568, 270-568, 283-568, 308-568, 393-568 | NM_006640 | Cell cycle, cell division, heterooligomerization | Osaka, *et al,* 1999 |
| Septin 7 (SEPT7) | **22** / 08 / 14 | (436)  11-400, 11-436, 23-436, 25-400 | NM_001788 | Cell cycle, cell division, cytokinesis, heterooligomerization | Nakatsuru, et al, 1994 |
| Septin 4 (SEPT4) | **09** / 08 / 01 | (459)  1-250, 1-300, 58-260, 60-290, 120-478 | NM_080416 | Cell cycle, cytokinesis, regulation of apoptosis | Xie, *et al*, 1999 |
| Septin 2 (SEPT2) | **06** / 0 / 06 | (361)  1-361 | NM_004404 | Cell cycle, cell division,  [mitosis](http://www.ncbi.nlm.nih.gov/entrez/utils/fref.fcgi?http://amigo.geneontology.org/cgi-bin/amigo/go.cgi?view=details&depth=1&query=7067), [regulation of protein localization](http://www.ncbi.nlm.nih.gov/entrez/utils/fref.fcgi?http://amigo.geneontology.org/cgi-bin/amigo/go.cgi?view=details&depth=1&query=32880) | Mori, *et al*, 1996 |
| Septin 5 (SEPT5) | **05** / 05 / 0 | (369)  1-350, 4-350 | NM_002688 | Cell cycle, cytokinesis, regulation of exocytosis, synaptic vesicle targeting | Yagi, *et al*, 1994 |
| Septin 1 (SEPT1) | **03** / 0 / 03 | (367)  1-367 | NM_052838 | Cell cycle, cell division | Mori, et al, 1996 |
| complement component 1, q subcomponent binding protein (C1QBP) | **08**/ 03/ 05 | (282)  3-282, 7-282,  28-282, 71-282,  82-282 | NM_001212 | Immune response, protein binding | Busby, *et al*, 1990 |
| Centromere protein F (CENPF) | **03**/ 03/ 0 | (3114)  2773-3114 | NM_016343 | Cell division, cell proliferation, kinetochore assembly, response to drug | Testa, *et al*, 1994 |
| Cerebral endothelial cell adhesion molecule (CERCAM) | **05**/ 05/ 0 | (595)  3-160 | NM_016174 | Cell adhesion, [cellular component movement](http://www.ncbi.nlm.nih.gov/entrez/utils/fref.fcgi?http://amigo.geneontology.org/cgi-bin/amigo/go.cgi?view=details&depth=1&query=6928) | Starzyk, et al, 2000 |
| Cell cycle associated protein 1 (CAPRIN1) | **01**/ 0/ 01 | (709)  140-200 | NM_005898 | Cell cycle | Ellis, *et al*, 1995 |
| Endoplasmic reticulum protein 29 (ERP29) | **01/** 0/ 01 | (261)  1-261 | NM_006817 | Intracellular protein transport, protein secretion, protein folding | Hochstrasser, et al, 1992 |
| Family with sequence similarity 89, member B (FAM89B) | **01/** 0/ 01 | (189)  102-189 | NM_001098785 | ? | Golovkin, *et al*, 1998 |
| Histone deacetylase 11 (HDAC11) | **01**/0/ 01 | (347)  181-211 | NM_024827 | Transcription, histone deacetylation, chromatin modification | Gao, *et al*, 2002 |
| Kinesin family member 14 (KIF14) | **01**/ 0/ 01 | (1648)  139-351 | NM_014875 | Microtubule-based movement, translation | Nakagawa, *et al*, 1997 |
| Lamin B1 (LMNB1) | **01**/ 0/01 | (586)  322-381 | MN_005573 | Mitosis, [protein binding](http://www.ncbi.nlm.nih.gov/entrez/utils/fref.fcgi?http://amigo.geneontology.org/cgi-bin/amigo/go.cgi?view=details&depth=1&query=5515), [structural molecule activity](http://www.ncbi.nlm.nih.gov/entrez/utils/fref.fcgi?http://amigo.geneontology.org/cgi-bin/amigo/go.cgi?view=details&depth=1&query=5198) | Lin and Woman, 1995 |
| Lymphocyte cytosolic protein 1 (LCP1, plastin-2) | **01**/ 0/ 01 | (627)  1-240 | NM_002298.2 | [actin filament bundle assembly](http://www.ncbi.nlm.nih.gov/entrez/utils/fref.fcgi?http://amigo.geneontology.org/cgi-bin/amigo/go.cgi?view=details&depth=1&query=51017), [regulation of intracellular protein transport](http://www.ncbi.nlm.nih.gov/entrez/utils/fref.fcgi?http://amigo.geneontology.org/cgi-bin/amigo/go.cgi?view=details&depth=1&query=33157) | Lin, *et al*, 1988 |
| Pre-B-cell leukemia homeobox interacting protein 1 (PBXIP1) | **01**/ 0/ 01 | (731)  100-300 | NM_020524.2 | Cell differenciation, multicellular organismal development, negative regulation of transcription | Epplen and Epplen, 1994 |
| Protein inhibitor of activated STAT, 3 (PIAS3) | **01**/ 01/ 0 | (628)  110-460 | NM_006099 | [regulation of protein sumoylation](http://www.ncbi.nlm.nih.gov/entrez/utils/fref.fcgi?http://amigo.geneontology.org/cgi-bin/amigo/go.cgi?view=details&depth=1&query=33235), [transcription](http://www.ncbi.nlm.nih.gov/entrez/utils/fref.fcgi?http://amigo.geneontology.org/cgi-bin/amigo/go.cgi?view=details&depth=1&query=45449) | Chung, *et al*, 1997 |
| SH2B adaptor protein 3 (SH2B3) | **01**/ 0/ 01 | (575)  532-560 | NM_005475.2 | Cell differenciation, embryonic hemopoiesis, intracellular signaling cascade | Motto, *et al*, 1996 |
| SWI/SNF related, matrix associated, actin dependent regulator of chromatin, subfamily c, member 2 (SMARCC2) | **01**/ 0/ 01 | (1214)  604-890 | NM_139067 | Transcription, chromatin assembly or disassembly, chromatin remodeling | Wang, *et al*, 1996 |
| Ubiquitin fusion degradation 1 like (yeast) (UFD1L) | **01**/ 0/ 01 | (307)  1-307 | NM_005659 | Skeletal development, ubiquitin cycle, ubiquitin-dependent protein catabolic process | Pizzuti, *et al*, 1997 |
| Zinc finger protein 451  (ZNF451) | **01**/ 0/ 01 | (1061)  1-272 | NM_001726133 | [Regulation of transcription](http://www.ncbi.nlm.nih.gov/entrez/utils/fref.fcgi?http://amigo.geneontology.org/cgi-bin/amigo/go.cgi?view=details&depth=1&query=45449) | Adams, et al, 2005 |

( ⇑ septin 8 bait continued ⇑ )

**Septin 9, full-length protein, bait**

| Protein name/ abreviation | Number of clones  **Total**/HFB/ LEU | Coded protein residues | Acession number | Function | Protein domains (in retrieved regions) | Reference |
| --- | --- | --- | --- | --- | --- | --- |
| Septin 6 (SEPT6) | **05**/- / 05 | (427)  8-427  (429)  8-429  (434)  8-434 | NM_145802  NM_145800  NM_015129 | Cell cycle, cytokinesis | Full length  NGC | Kinoshita, *et al*, 2000 |
| Septin 7 (SEPT7) | **03** / - / 03 | (437)  11-437  (436)  5-436, 11-436 | NM_001788  NM_001011553 | Cell cycle, cell division, cytokinesis, | Full length  NGC | Nakatsuru, *et al*, 1994 |

**Septin 9, N-terminal region only (amino acids 1-269), bait**

| Protein name/ abreviation | Number of clones  **Total**/HFB/ LEU | Coded protein residues | Acession number | Function | Protein domains (in retrieved regions) | Reference |
| --- | --- | --- | --- | --- | --- | --- |
| Filamin A (FLNA) | 03/ - /03 | 2647)  2162-2647  (2639)  1104-2639  (2612)  908-2612 | NM_001110556  NM_001456  AB371579 | actin cytoskeleton organization, scaffold, anchor for transmembrane proteins | Filamin-type immunoglobulin domains | van der Flier and Sonnenberg, 2001. |
| SH3-domain kinase binding protein 1 (SH3KBP1 / HSB1 / CIN85 / GIG10 / HSB-1 / MIG18 / CD2BP3) | 02/ - /02 | (665)  11-665  (628)  8-628 | NM_031892  NM_001024666 | cytoskeleton remodeling, vesicle-mediated transport, signal transduction, cell death | SH3 domains | Havrylov *et al.*, 2010 |

**Septin 10 bait**

| Protein name/ abreviation | Number of clones  **Total**/HFB/ LEU | Coded protein residues | Acession number | Function | Reference |
| --- | --- | --- | --- | --- | --- |
| zinc finger and BTB domain containing 16 (ZBTB16) | **03** / 03 / - | (673)  20 - 673  152 -673  290 - 673 | NM_006006 | cell cycle progression, apoptosis | Chen, *et al*, 1993 |

**References cites in Table S1:**

Adams MD, Kerlavage AR, Fleischmann RD, Fuldner RA, Bult CJ et al. (1995) Initial assessment of human gene diversity and expression patterns based upon 83 million nucleotides of cDNA sequence. Nature 377: 3-174.

Alderuccio F, Chan EK, Tan EM (1991) Molecular characterization of an autoantigen of PM-Scl in the polymyositis/scleroderma overlap syndrome: a unique and complete human cDNA encoding an apparent 75-kD acidic protein of the nucleolar complex. J. Exp. Med. 173: 941-952.

Allikmets R, Gerrard B, Glavac D, Ravnik-Glavac M, Jenkins NA et al. (1995) Characterization and mapping of three new mammalian ATP-binding transporter genes from an EST database. Mamm. Genome 6: 114-117.

# [**Begley DA**](http://www.ncbi.nlm.nih.gov/pubmed?term="Begley DA"%5BAuthor%5D), [**Berkenpas MB**](http://www.ncbi.nlm.nih.gov/pubmed?term="Berkenpas MB"%5BAuthor%5D), [**Sampson KE**](http://www.ncbi.nlm.nih.gov/pubmed?term="Sampson KE"%5BAuthor%5D), [**Abraham I**](http://www.ncbi.nlm.nih.gov/pubmed?term="Abraham I"%5BAuthor%5D) (1997) Identification and sequence of human PKY, a putative kinase with increased expression in multidrug-resistant cells, with homology to yeast protein kinase Yak1. [**Gene**](javascript:AL_get(this, 'jour', 'Gene.');) 200: 35-43.

Bläser S, Jersch K, Hainmann I, Zieger W, Wunderle D, et al. (2003) Isolation of new splice isoforms, characterization and expression analysis of the human septin SEPT8 (KIAA0202). Gene 312: 313-320.

Brown SJ, Jewell A, Maki CG, Roufa DJ (1990) A cDNA encoding human ribosomal protein S24. Gene 91: 293-296.

Chen Z, Brand NJ, Chen A, Chen SJ, Tong JH et al. (1993) Fusion between a novel Kruppel-like zinc finger gene and the retinoic acid receptor-alpha locus due to a variant t(11;17) translocation associated with acute promyelocytic leukaemia. EMBO J. 12: 1161-1167.

Chung CD, Liao J, Liu B, Rao X, Jay P et al. (1997) Specific inhibition of Stat3 signal transduction by PIAS3. Science 278: 1803-1805.

Cortázar D, Kunz C, Saito Y, Steinacher R, Schär P (2007) The enigmatic thymine DNA glycosylase. DNA Repair 6: 489-504.

Dunham I, Shimizu N, Roe BA, Chissoe S, Hunt AR et al. (1999) [The DNA sequence of human chromosome 22.](http://www.ncbi.nlm.nih.gov/pubmed/10591208) Nature 402: 489-95.

Epplen C, Epplen JT (1994) Expression of (cac)n/(gtg)n simple repetitive sequences in mRNA of human lymphocytes. Hum. Genet. 93: 35-41.

Fukuyama K, Yoshida M, Yamashita A, Deyama T, Baba M et al. (2000) MAPK upstream kinase (MUK)-binding inhibitory protein, a negative regulator of MUK/dual leucine zipper-bearing kinase/leucine zipper protein kinase. J. Biol. Chem. 275: 21247-21254.

Gao L, Cueto MA, Asselbergs F, Atadja, P (2002) Cloning and functional characterization of HDAC11, a novel member of the human histone deacetylase family. J. Biol. Chem. 277: 25748-25755.

Golovkina TV, Dzuris J, van den Hoogen B, Jaffe AB, Wright PC et al. (1998) A novel membrane protein is a mouse mammary tumor virus receptor. J. Virol. 72: 3066-3071.

[Haluska P Jr](http://www.ncbi.nlm.nih.gov/pubmed?term="Haluska P Jr"%5BAuthor%5D), [Saleem A](http://www.ncbi.nlm.nih.gov/pubmed?term="Saleem A"%5BAuthor%5D), [Rasheed Z](http://www.ncbi.nlm.nih.gov/pubmed?term="Rasheed Z"%5BAuthor%5D), [Ahmed F](http://www.ncbi.nlm.nih.gov/pubmed?term="Ahmed F"%5BAuthor%5D), [Su EW](http://www.ncbi.nlm.nih.gov/pubmed?term="Su EW"%5BAuthor%5D) et al. (1999) Interaction between human topoisomerase I and a novel RING finger/arginine-serine protein. 27: 2538-2544.

Hanai K, Nagata A, Kawajiri T, Shiromizu N, Saitoh et al. (2004) Biochemical and cell biological characterization of a mammalian septin, Sept11. FEBS Letters 568: 83 – 88.

[Hanisch A](http://www.ncbi.nlm.nih.gov/pubmed?term="Hanisch A"%5BAuthor%5D), [Silljé HH](http://www.ncbi.nlm.nih.gov/pubmed?term="Silljé HH"%5BAuthor%5D), [Nigg EA](http://www.ncbi.nlm.nih.gov/pubmed?term="Nigg EA"%5BAuthor%5D) (2006) Timely anaphase onset requires a novel spindle and kinetochore complex comprising Ska1 and Ska2. [EMBO Journal](javascript:AL_get(this, 'jour', 'EMBO %0D%0AJ.');) 25: 5504-15.

Havrylov S, Redowicz MJ, Buchman VL (2010) Emerging roles of Ruk/CIN85 in vesicle-mediated transport, adhesion, migration and malignancy. Traffic 11: 721-731.

Hochstrasser DF, Frutiger S, Paquet N, Bairoch A, Ravier F et al. (1992) Human liver protein map: a reference database established by microsequencing and gel comparison. Electrophoresis 13: 992-1001.

Imai Y, Kimura T, Murakami A, Yajima N, Sakamaki,K et al. (1999) The CED-4-homologous protein FLASH is involved in Fas-mediated activation of caspase-8 during apoptosis. Nature 398: 777-785.

Jullien-Flores V, Dorseuil O, Romero F, Letourneur F, Saragosti S et al. (1995) GTPase to Rho pathways. RLIP76, a Ral effector with CDC42/Rac GTPase-activating protein activity. Journal of Biological Chemistry, 270: 22473-22477

Karvonen U, Jaaskelainen T, Rytinki M, Kaikkonen S, Palvimo JJ (2008) ZNF451 is a novel PML body- and SUMO-associated transcriptional coregulator. Journal Molelucar Biology 382: 585-600

Kauselmann G, Weiler M, Wulff P, Jessberger S, Konietzko U et al. (1999) The polo-like protein kinases Fnk and Snk associate with a Ca(2+)- and integrin-binding protein and are regulated dynamically with synaptic plasticity. EMBO J. 18: 5528-5539.

Kinoshita M, Kumar S, Mizoguchi A, Ide C, Kinoshita A et al. (1997) Nedd5, a mammalian septin, is a novel cytoskeletal component interacting with actin-based structures. Genes & Development 11: 1535-1547.

Kinoshita A, Noda M, Kinoshita M (2000) Differential localization of septins in the mouse brain. J. Comp. Neurol. 428: 223-239.

[Larson LM](http://www.ncbi.nlm.nih.gov/pubmed?term="Larson LM"%5BAuthor%5D), [Bruce AW](http://www.ncbi.nlm.nih.gov/pubmed?term="Bruce AW"%5BAuthor%5D), [Saumur JH](http://www.ncbi.nlm.nih.gov/pubmed?term="Saumur JH"%5BAuthor%5D), [Wasdahl WA](http://www.ncbi.nlm.nih.gov/pubmed?term="Wasdahl WA"%5BAuthor%5D) (1982) Further evidence by gene dosage for the regional assignment of erythrocyte acid phosphatase (ACP1) and malate dehydrogenase (MDH1) loci on chromosome 2p. Clin Genet. 22: 220-225.

Li SH, McInnis MG, Margolis RL, Antonarakis SE, Ross CA (1993) Novel triplet repeat containing genes in human brain: cloning, expression, and length polymorphisms. Genomics 16: 572-579.

Lin CS, Aebersold RH, Kent SB, Varma M, Leavitt J (1988) Molecular cloning and characterization of plastin, a human leukocyte protein expressed in transformed human fibroblasts Mol. Cell. Biol. 8: 4659-4668.

Lin F, Worman HJ (1995) Structural organization of the human gene (LMNB1) encoding nuclear lamin B1. Genomics 27: 230-236.

Matsushime H, Yoshida MC, Sasaki M, Shibuya, M (1987) A possible new member of tyrosine kinase family, human frt sequence, is highly conserved in vertebrates and located on human chromosome 13. Jpn. J. Cancer Res. 78: 655-661.

Mori T, Miura K, Fujiwara T, Shin S, Inazawa J et al. (1996) Isolation and mapping of a human gene (DIFF6) homologous to yeast CDC3, CDC10, CDC11, and C spindle and kinetochore-associated protein 2 isoform 1 DC12, and mouse Diff6. Cytogenetics and Cell Genetics 73: 224-227.

Motto DG, Musci MA, Ross SE, Koretzky, GA (1996) Tyrosine phosphorylation of Grb2-associated proteins correlates with phospholipase C gamma 1 activation in T cells. Mol. Cell. Biol. 16: 2823-2829.

# [**Nakagawa T**](http://www.ncbi.nlm.nih.gov/pubmed?term="Nakagawa T"%5BAuthor%5D), [**Tanaka Y**](http://www.ncbi.nlm.nih.gov/pubmed?term="Tanaka Y"%5BAuthor%5D), [**Matsuoka E**](http://www.ncbi.nlm.nih.gov/pubmed?term="Matsuoka E"%5BAuthor%5D), [**Kondo S**](http://www.ncbi.nlm.nih.gov/pubmed?term="Kondo S"%5BAuthor%5D), [**Okada Y**](http://www.ncbi.nlm.nih.gov/pubmed?term="Okada Y"%5BAuthor%5D) (1997) Identification and classification of 16 new kinesin superfamily (KIF) proteins in mouse genome. [**Proc Natl Acad Sci U S A**](javascript:AL_get(this, 'jour', 'Proc Natl Acad Sci U S A.');) 94: 9654-9659.

Nakatsuru S, Sudo K, Nakamura Y (1994) [Molecular cloning of a novel human cDNA homologous to CDC10 in Saccharomyces cerevisiae.](http://www.ncbi.nlm.nih.gov/pubmed/8037772) Biochem Biophys Res Commun 202: 82-7.

Oh, JH, Yang JO, Hahn Y, Kim MR, Byun SS et al. (2005) Transcriptome analysis of human gastric cancer. Mamm. Genome 16: 942-954.

Ono R, Taki T, Taketani T, Kawaguchi H, Taniwaki M et al. (2002) SEPTIN6, a human homologue to mouse Septin6, is fused to MLL in infant acute myeloid leukemia with complex chromosomal abnormalities involving 11q23 and Xq24. Cancer Research 62: 333-337.

[Osaka M](http://www.ncbi.nlm.nih.gov/sites/entrez?Db=pubmed&Cmd=Search&Term="Osaka M"%5BAuthor%5D&itool=EntrezSystem2.PEntrez.Pubmed.Pubmed_ResultsPanel.Pubmed_DiscoveryPanel.Pubmed_RVAbstractPlus), [Rowley JD](http://www.ncbi.nlm.nih.gov/sites/entrez?Db=pubmed&Cmd=Search&Term="Rowley JD"%5BAuthor%5D&itool=EntrezSystem2.PEntrez.Pubmed.Pubmed_ResultsPanel.Pubmed_DiscoveryPanel.Pubmed_RVAbstractPlus), [Zeleznik-Le NJ](http://www.ncbi.nlm.nih.gov/sites/entrez?Db=pubmed&Cmd=Search&Term="Zeleznik-Le NJ"%5BAuthor%5D&itool=EntrezSystem2.PEntrez.Pubmed.Pubmed_ResultsPanel.Pubmed_DiscoveryPanel.Pubmed_RVAbstractPlus) (1999) MSF (MLL septin-like fusion), a fusion partner gene of MLL, in a therapy-related acute myeloid leukemia with a t(11;17)(q23;q25). [Proc Natl Acad Sci](javascript:AL_get(this, 'jour', 'Proc Natl Acad Sci U S A.');) USA 96: 6428-6433.

Okazaki T, Wang H, Masliah E, Cao M, Johnson SA et al. (1995) SCG10, a neuron-specific growth-associated protein in Alzheimer's disease. Neurobiol. Aging 16: 883-894.

Parks WT, Frank DB, Huff C, Renfrew Haft C et al. (2001) Sorting nexin 6, a novel SNX, interacts with the transforming growth factor-beta family of receptor serine-threonine kinases. The Journal of Biological Chemistry 276: 19332-19339.

Pizzuti A, Novelli G, Ratti A, Amati F, Mari A et al. (1997) UFD1L, a developmentally expressed ubiquitination gene, is deleted in CATCH 22 syndrome Hum. Mol. Genet. 6: 259-265.

Rasmussen HH, van Damme J, Puype M, Gesser B, Celis JE et al. (1992) Microsequences of 145 proteins recorded in the two-dimensional gel protein database of normal human epidermal keratinocytes. Electrophoresis 13: 960-969.

Reddy NS, Roth WW, Bragg PW, Wahba AJ (1988) Isolation and mapping of a gene for protein synthesis initiation factor 4A and its expression during differentiation of murine erythroleukemia cells. Gene 70: 231-243.

Ruppert C, Kroschewski R, Bahler M (1993) Identification, characterization and cloning of myr 1, a mammalian myosin-I. J. Cell Biol. 120: 1393-1403.

Qin H, Wang Z, Diener D, Rosenbaum J (2007) Intraflagellar transporta protein 27 is a small G protein involved in cell-cycle control. Current Biology 17: 193-202.

Sagiv Y, Legesse-Miller A, Porat A, Elazar Z (2000) GATE-16, a membrane transport modulator, interacts with NSF and the Golgi v-SNARE GOS-28. EMBO J. 19: 1494-1504.

Shen Z, Pardington-Purtymun PE, Comeaux JC, Moyzis RK, Chen DJ (1996) UBL1, a human ubiquitin-like protein associating with human RAD51/RAD52 proteins Genomics 36: 271-279.

Starzyk RM, Rosenow C, Frye J, Leismann M, Rodzinski,E et al. (2000) Cerebral cell adhesion molecule: a novel leukocyte adhesion determinant on blood-brain barrier capillary endothelium. J. Infect. Dis. 181: 181-187.

Sui L, Zhang W, Liu Q, Chen T, Li N et al. (2003) Cloning and functional characterization of human septin 10, a novel member of septin family cloned from dendritic cells, Biochem. Biophys. Res. Commun. 304: 393-398.

Tomasetto C, Regnier C, Moog-Lutz C, Mattei MG, Chenard MP et al. (1995) Identification of four novel human genes amplified and overexpressed in breast carcinoma and localized to the q11-q21.3 region of chromosome 17. Genomics 28: 367-376.

van der Flier A., Sonnenberg A (2001) Structural and functional aspects of filamins. Biochimica et Biophysica Acta. 1538: 99-117.

Vandekerckhove J, Weber K (1978) Mammalian cytoplasmic actins are the products of at least two genes and differ in primary structure in at least 25 identified positions from skeletal muscle actins. Proc. Natl. Acad. Sci. USA. 75: 1106-1110.

Venter JC, Adams MD, Myers EW, Li PW, Mural RJ et al. (2001) The sequence of the human genome. Science 291: 1304-1351.

Wada M, Ren CH, Koyama S, Arawaka S, Kawakatsu S et al. (2004) A human granin-like neuroendocrine peptide precursor (proSAAS) immunoreactivity in tau. inclusions of Alzheimer's disease and parkinsonism-dementia complex on Guam Neurosci. Lett. 356: 49-52.

Wakamatsu A, Yamamoto J, Kimura K, Ishii S, Watanabe K et al. NEDO human cDNA sequencing project. Unpublished database entry

Wang W, Xue Y, Zhou S, Kuo A, Cairns BR (1996) Crabtree GR.[Diversity and specialization of mammalian SWI/SNF complexes.](http://www.ncbi.nlm.nih.gov/pubmed/8804307) Genes Dev. 1996: 10: 2117-2130.

Watanabe TK, Fujiwara T, Kawai A, Shimizu F, Takami S et al. (1996) Cloning, expression, and mapping of UBE2I, a novel gene encoding a human homologue of yeast ubiquitin-conjugating enzymes which are critical for regulating the cell cycle. Cytogenetics and Cell Genetics 72: 86-89.

[Welch MD](http://www.ncbi.nlm.nih.gov/pubmed?term="Welch MD"%5BAuthor%5D), [Iwamatsu A](http://www.ncbi.nlm.nih.gov/pubmed?term="Iwamatsu A"%5BAuthor%5D), [Mitchison TJ](http://www.ncbi.nlm.nih.gov/pubmed?term="Mitchison TJ"%5BAuthor%5D). (1997) Actin polymerization is induced by Arp2/3 protein complex at the surface of Listeria monocytogenes. [Nature](javascript:AL_get(this, 'jour', 'Nature.');) 385: 265-269.

Xie H, Surka M, Howard J, Trimble WS (1999) Characterization of the mammalian septin H5: distinct patterns of cytoskeletal and membrane association from other septin proteins. Cell Motil. Cytoskeleton 43: 52-62.

Yagi M, Zieger B, Roth GJ, Ware J (1998) Structure and expression of the human septin gene HCDCREL-1. Gene 212: 229-236.

**Ou YY, Mack GJ, Zhang M, Rattner JB (2002)** CEP110 and ninein are located in a specific domain of the centrosome associated with centrosome maturation. Journal of Cell Science 115: 1825-1835.

Zhang A, Yeung PL, Li CW, Tsai SC, Dinh GK et al. (2004) Identification of a novel family of ankyrin repeats containing cofactors for p160 nuclear receptor coactivators. Journal of Biological Chemistry 279: 33799-33805.

**Results of the statistical analysis**

# 1+2+4+5 6+8+10+11 TotCol

# 1+2+4+5 10 53 63

# 6+8+10+11 52 0 52

# TotLin 62 53 115

#

# Fisher's Exact Test for Count Data

# P value = 0, Diagnostic: (P <= 0.001) SIGNIFICATant

# alternate hypothesis: Odds Ratio not equal to 1 (two.sided)

# Odds Ratio = 0

# Interval of confidence (95%) = 0 a 0.02

# 1+2+4+5 3+9 TotCol

# 1+2+4+5 10 0 10

# 3+9 15 0 15

# TotLin 25 0 25

#

# Fisher's Exact Test for Count Data

# P value = 1, Diagnostic: not significant

# alternate hypothesis: Odds Ratio not equal to 1 (two.sided)

# Odds Ratio = 0

# confidence interval (95%) = 0 a Inf

# 1+2+4+5 7 TotCol

# 1+2+4+5 10 4 14

# 7 0 0 0

# TotLin 10 4 14

#

# Fisher's Exact Test for Count Data

# P value = 1, Diagnostic: not significant

# alternate hypothesis: Odds Ratio note qual to 1 (two.sided)

# Odds Ratio = 0

# confidence Interval (95%) = 0 a Inf

# 6+8+10+11 3+9 TotCol

# 6+8+10+11 0 16 16

# 3+9 146 0 146

# TotLin 146 16 162

#

# Fisher's Exact Test for Count Data

# P value = 0, Diagnostic: (P <= 0.001) significant

# alternate hypothesis: Odds Ratio note qual to 1 (two.sided)

# Odds Ratio = 0

# confidence Interval(95%) = 0 a 0

# 6+8+10+11 7 TotCol

# 6+8+10+11 0 13 13

# 7 31 0 31

# TotLin 31 13 44

#

# Fisher's Exact Test for Count Data

# P value = 0, Diagnostic: (P <= 0.001) SIGNIFICAnt

# alternate Hypothesis: Odds Ratio note qual to 1 (two.sided)

# Odds Ratio = 0

# confidence interval (95%) = 0 a 0.02

# 3+9 7 TotCol

# 3+9 0 34 34

# 7 3 0 3

# TotLin 3 34 37

#

# Fisher's Exact Test for Count Data

# P value = 0, Diagnostic: (P <= 0.001) SIGNIFICant

# alternate hypothesis: Odds Ratio note qual to 1 (two.sided)

# Odds Ratio = 0

# confidence Interval (95%) = 0 a 0.11

Whole table:

$porc.tot

1 1 2 4 5 6 8 10 11 3 9 7 TotCol

0.80% 0.00% 0.00% 0.00% 2.12% 0.80% 0.00% 0.00% 0.00% 0.00% 0.27% 3.98%

2 0.53% 0.00% 0.00% 0.27% 1.33% 1.59% 0.00% 0.00% 0.00% 0.00% 0.80% 4.51%

4 0.27% 0.27% 0.00% 0.00% 1.33% 2.39% 0.00% 0.00% 0.00% 0.00% 0.00% 4.24%

5 0.27% 0.00% 0.00% 0.27% 3.18% 1.33% 0.00% 0.00% 0.00% 0.00% 0.00% 5.04%

6 3.71% 1.33% 0.53% 2.12% 0.00% 0.00% 0.00% 0.00% 1.59% 1.33% 2.92% 13.53%

8 0.00% 0.00% 0.27% 1.59% 0.00% 0.00% 0.00% 0.00% 0.00% 0.00% 0.00% 1.86%

10 0.00% 0.00% 0.80% 0.00% 0.00% 0.00% 0.00% 0.00% 0.00% 0.00% 0.27% 1.06%

11 0.80% 0.00% 1.86% 0.80% 0.00% 0.00% 0.00% 0.00% 1.33% 0.00% 0.27% 5.04%

3 0.00% 0.00% 0.00% 0.00% 0.27% 0.00% 0.00% 0.00% 0.00% 0.00% 0.00% 0.27%

9 3.98% 0.00% 0.00% 0.00% 25.46% 13.00% 0.00% 0.00% 0.00% 0.00% 9.02% 51.46%

7 0.00% 0.00% 0.00% 0.00% 2.39% 5.84% 0.00% 0.00% 0.00% 0.80% 0.00% 9.02%

TotLin

10.34% 1.59% 3.45% 5.04% 36.07% 24.93% 0.00% 0.00% 2.92% 2.12% 13.53% 100.00%

fisher.test (sept, simulate.p.value = T)

Fisher's Exact Test for Count Data with simulated p-value (based on 2000 replicates)

data: sept

p-value = 0.0004998

alternative hypothesis: two.sided
