# Supplementary material for: A Draft of the Human Septin Interactome
Source: PLoS One. 2010 Nov 2;5(11):e13799. doi: 10.1371/journal.pone.0013799 (PMC2970546; doi:10.1371/journal.pone.0013799)
Supplement: Figure S1 — A protein interaction network of the human septins 1-10. The network consists of a total of proteins (colored nodes, including the septin baits and its interacting partners identified in the yeast two-hybrid screens) and the interactions connecting them (grey links). The nodes are colored based on the GO biological process as indicated in the legend. The network was generated using the Osprey 1.2.0. software (http://biodata.mshri.on.ca/osprey/). The proteins that interacted with septin are involved in Carbohydrate Metabolism, Cell Cycle, Cell Organization and Biogenesis, DNA Damage Response, DNA metabolism, DNA Repair, Metabolism, Protein amino acid phosphorylation, Protein biosynthesis, Protein transport, RNA Localization, RNA processing, Signal transduction, Transcription, Transport. (0.11 MB DOC) [file pone.0013799.s002.doc]

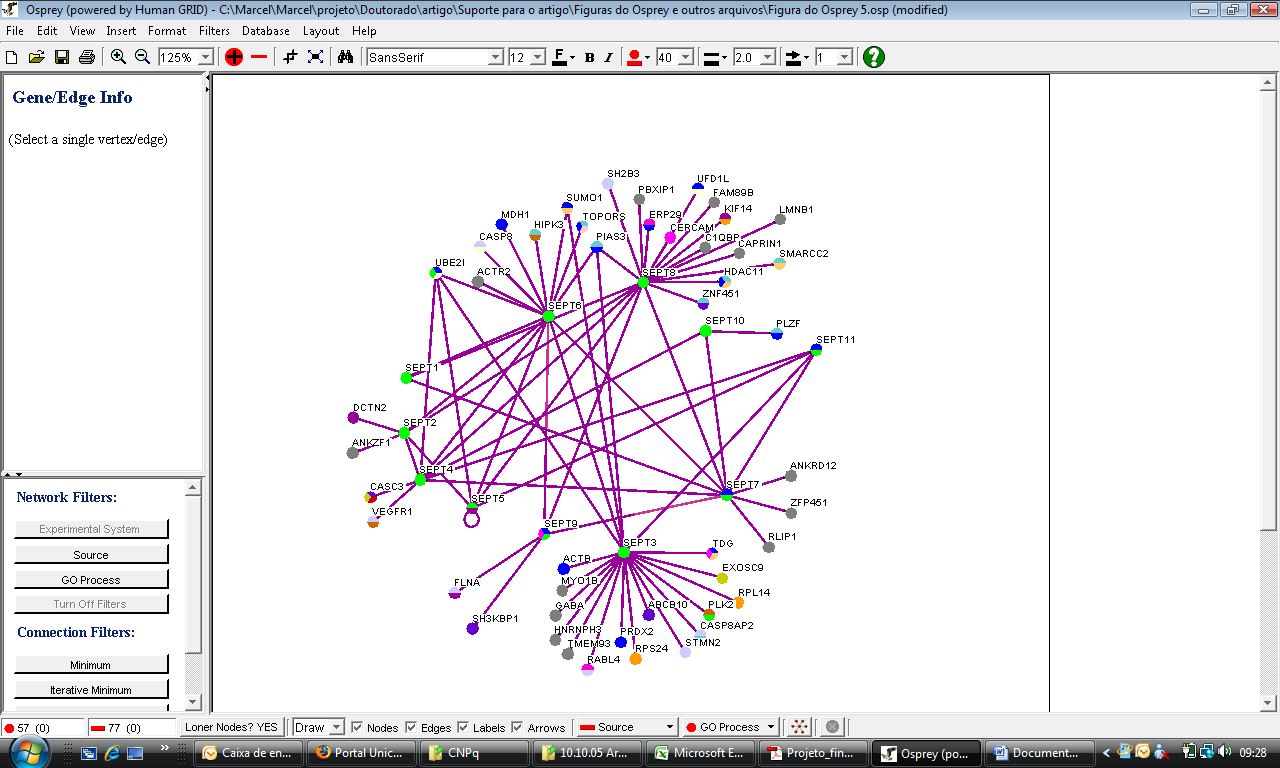


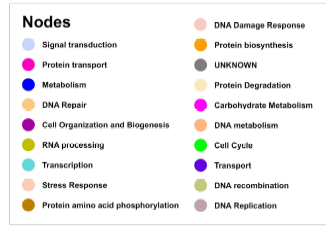


**Supplemental figure S1: A protein interaction network of the human septins 1-10.** The network consists of a total of proteins (colored nodes, including the septin baits and its interacting partners identified in the yeast two-hybrid screens) and the interactions connecting them (grey links). The nodes are colored based on the GO biological process as indicated in the legend. The network was generated using the Osprey 1.2.0. software (http://biodata.mshri.on.ca/osprey/). The proteins that interacted with septin are involved in Carbohydrate Metabolism , Cell Cycle, Cell Organization and Biogenesis, DNA Damage Response, DNA metabolism, DNA Repair, Metabolism, Protein amino acid phosphorylation, Protein biosynthesis, Protein transport, RNA Localization, RNA processing, Signal transduction, Transcription, Transport.
